# Examining the mental health support needs of NHS staff exposed to patient pregnancy and neonatal loss: A qualitative study

**DOI:** 10.1371/journal.pone.0358321

**Published:** 2026-09-17

**Authors:** Debbie Johnson, Sarah Newell, Hannah Family, Madeleine Holland, Maggie Westby, Eleanor Franks, Laura Turner, China Harrison

**Affiliations:** 1 Population Health Sciences, Bristol Medical School, University of Bristol, Bristol, United Kingdom; 2 University Hospitals Bristol and Weston, Bristol United Kingdom; 3 National Institute for Health and Care Research Applied Research Collaboration West (NIHR ARC West), Bristol, United Kingdom; 4 National Institute for Health and Care Research, Health Protection Research Unit (HPRU) in Evaluation and Behavioural Science (EBS), Bristol, United Kingdom; 5 School of Healthcare, University of Leicester, Leicester, United Kingdom; 6 Desitin Pharmaceuticals Ltd, Fairbourne Drive, Milton Keynes, United Kingdom; University of Education Winneba Faculty of Science Education, GHANA

## Abstract

**Background:**

Pregnancy and neonatal loss, including miscarriage, stillbirth, termination for fetal abnormality, and neonatal death, affects a substantial proportion of pregnancies in the UK. Although the impact on patients is well documented, less attention has been paid to the experiences of NHS staff providing care during these events.

**Study aims:**

To explore staff experiences of exposure to pregnancy and neonatal loss, perceptions of available support, support previously used, and needed or wanted.

**Methods:**

This qualitative study used a reflective thematic approach. Semi-structured interviewed were conducted with 20 purposefully sampled clinical, allied and non-clinical staff from a single UK NHS trust. Interviews were audio-recorded, transcribed verbatim, anonymised and analysed using thematic analysis, with themes mapped to the study aims. Results: Six themes were identified: (1) nobody prepared me for this (2) reaching a tipping point; (3) peer support versus inconsistent organisational support; (4) reliance on informal support networks; (5) bereavement team and multi-disciplinary support buffer the stressful effects of loss; and (6) one size does not fit all. Exposure varied in type, frequency, and intensity across roles. Many staff, particularly junior, allied and support staff, felt underprepared for the emotional and communication challenges of pregnancy and neonatal loss. Participants described reaching a “tipping point” where resilience was compromised. Peer support was the most accessible and reliable resource, whereas organisational support was underused despite being valued.

**Conclusion:**

The emotional demands experienced by NHS staff are inconsistently supported. Findings highlight a “tipping point” where cumulative exposure to pregnancy and neonatal loss can overwhelm coping capacity, particularly among staff in junior, allied and support roles who often report lower levels of preparation and access to formal support. A proactive multi-disciplinary approach is needed to provide visible and equitable support tailored to diverse staff needs.

## Introduction

Pregnancy and neonatal loss, encompassing miscarriage, stillbirth, termination for fetal abnormality, and neonatal death, affects a substantial proportion of pregnancies in the United Kingdom (UK). Miscarriage alone is estimated to occur in one in four recognised pregnancies, while around 1 in 250 births results in stillbirth and thousands of families each year experience neonatal death or termination for fetal abnormality [1]. Although the impact on patients is well-documented [2,3], less attention has been given to the experiences of National Health Service (NHS) clinical (e.g., nurses, midwives, obstetricians, gynaecologists) allied (e.g., sonographers, theatre) and support staff (e.g., administrators) who provide care during these events [4–7].

A wide range of staff working in Obstetrics and Gynaecology (O&G), encounter pregnancy and neonatal loss as part of their day-to-day work. Such exposure has been associated with emotional distress, burnout, compassion fatigue, perceived professional failure, reduced job satisfaction and staff absence. Staff working in high-stress settings, such as Neonatal Intensive Care Units (NICUs) may be particularly vulnerable [8–10]. The relational nature of perinatal care, combined with repeated or unsupported exposure to loss, may heighten the risk of psychological trauma and secondary traumatic stress, especially for those with personal experiences of loss [11–14].

In the UK, supporting the mental health of NHS staff is highlighted in several national and regional policy initiatives. The NHS staff survey [15] and the Bristol, North Somerset, South Gloucestershire (BNSSG) mental health and wellbeing strategy [16] identify stress, anxiety, and depression as key contributors to staff sickness absence. Similarly, the Royal College of Midwives ‘Caring for You’ campaign highlights the need for improved mental health support for maternity staff [17]. Evidence also points to the value of targeted education, training and emotional support for staff [18].

Despite this, research on staff experiences remains limited, with much of the existing evidence focusing on clinical staff. Little attention has been given to allied and non-clinical staff who also encounter patient pregnancy and neonatal loss. Furthermore, there is a lack of research examining the availability and effectiveness of organisational support.

While support interventions such as debriefing, supervision and peer support exist, their availability and effectiveness remain inconsistent [19]. Consequently, further research is needed to examine mental health support needs, and to determine what support is most beneficial. This study aimed to examine: 1) experiences of exposure to pregnancy and neonatal loss; 2) perceived support available; 3) support previously used; and 4) support needed or wanted

## Materials and methods

### Design and setting

This qualitative study used a reflective thematic analysis approach to explore staff experiences of pregnancy and neonatal loss. Semi-structured individual interviews were carried out with staff working in a large NHS Trust in the UK. The Trust provides maternity, gynaecological, fetal medicine, neonatal and ultrasound services across two hospital sites and conducts around 5000 deliveries annually. It also provides tertiary maternal and fetal medicine care for patients across some of the most socio-economically deprived areas in England. This means that staff frequently provide complex, high-risk maternity and neonatal care and may be more likely to come across pregnancy and neonatal loss.

### Participants and sampling

Healthcare professionals, allied and support staff were eligible to participate if they were over 18 years of age and had interactions with patients experiencing pregnancy and neonatal loss as part of their role. A recruitment email was sent to all staff in relevant departments and recruitment posters were placed in staff areas from 20.07.2025 to 09.02.2026. Prospective participants could register their interest by completing a short online questionnaire or by contacting the research team directly.

Purposive sampling was used to arrange interviews with a diverse group of staff across departments and employment roles. Interviews took place across eight months to enable an iterative approach to each interview allowing both revision of the topic guide and further sampling of participants. As data collection progressed, emerging findings also informed decisions about recruitment, with additional staff groups identified as important to include in order to further explore developing themes and ensure adequate representation across relevant roles. For example, later interviews prompted further sampling of allied staff and refinement of interview prompts to explore areas such as preparedness and access to informal support in greater depth.

Given the remote interview format participants were asked to provided verbal informed consent prior to participation, which was approved by the National Health Service, Health Research Authority, Research Ethics Committee (25/HRA/2520 IRAS: 357165) and the University of Bristol Faculty of Health Sciences Research Ethics Committee (FREC) (REF: 25063), Participants were sent a participant information sheet in advance of the interview and had the opportunity to ask questions before consent was obtained. Verbal consent was obtained by DJ at the start of each interview and was audio-recorded as part of the interview recording, thereby providing a documented record of consent. Consent recordings were stored securely on encrypted institutional servers, separately from anonymised interview data. Participants were reimbursed with a shopping voucher as a thank you for their time.

The reporting of this study was guided by the Standards for Reporting Qualitative Research (SRQR). The completed SRQR checklist is provided as Supporting Information (S1 Checklist).

### Semi-structured interviews

Interviews were carried out online (MS Teams) by DJ (a research associate with a background in health research including topics related to pregnancy, breastfeeding and perinatal mental health in addition to previous midwifery experience). A flexible topic guide, informed by the aims of the study, was developed and adapted appropriately for the participant’s job role. Reflexive consideration was given to how DJs positioning may have influenced participant responses and interview dynamics.

### Data management and analysis

Interviews were audio recorded and transcribed verbatim by a professional transcription service. NVivo software (QSR International Pty Ltd.,) was used to manage and analyse data using thematic analysis [20] following an iterative interpretive and reflexive approach. Transcripts were read several times for familiarisation, and initial codes were generated inductively by DJ to capture meaningful features of the data. CH (a behavioural scientist with expertise in sexual and reproductive health) independently read and coded a substantial proportion of the transcripts, contributing alternative interpretations, supporting reflexivity and the development of a richer, more nuanced analysis. Throughout the analysis, DJ and CH regularly met with SN (Consultant Obstetrician and Subspecialist in Fetal/Maternal Medicine), MH (Lead Neonatal Nurse for Bereavement), HF (Psychologist and Behavioural Scientist with expertise in qualitative research) to reflect on assumptions and discuss analytic decisions while considering how differing perspectives and backgrounds may have shaped interpretation of the data. Sub-themes were developed iteratively through close engagement with the dataset and were generated from patterns identified across participant accounts rather than being predetermined by the study aims. Subthemes were further refined and organised into broader overarching themes. Deviating from Braun and Clarke’s approach to reporting findings the themes were organised in relation to the aims of the research to support coherent presentation and interpretation of the findings which are intended to inform future intervention development.

## Results

### Recruitment outcome

A total of 49 staff members volunteered to be interviewed with 20 purposefully recruited to participate in the interviews.

Interviews lasted between 42 and 84 minutes. Interviewees included: Doctors (Consultants, Residents and rotational) from O&G, Fetal Medicine Unit (FMU) and NICU (n = 5); Nurses and Nursing Assistants (Bands 3, 6 & 7) from Early Pregnancy Clinic (EPC), Gynaecology, and NICU(n = 6); Operating Theatre staff (n = 2) Sonographers (n = 2); Administration staff (n = 1) and Midwives (bands 5, 6 and 7) from continuity of carer team / rotational staff and Delivery Suite (n = 4).

### Exposure to loss

The type, frequency and intensity of exposure to patient pregnancy and neonatal loss varied by job role and department (see S1 Table). Staff working in Gynaecology, Sonography and FMU reported frequent exposure to early pregnancy loss, often managing multiple cases daily. In contrast, late-gestation or neonatal loss occurred less frequently and the larger midwifery workforce in the Delivery Suite enabled more careful allocation of cases to minimise repeated exposure. Midwives also appeared to have more flexibility to ‘opt out’ of caring for patients experiencing a loss. In comparison, sonographers and doctors across all grades reported greater and more frequent exposure to a wide range of pregnancy and neonatal loss scenarios, where their roles can involve direct management of more complex cases, terminations for fetal abnormalities and occasionally, feticide. The smaller number of medical staff also reported limited opportunities to step back from such cases, even when individuals feel emotionally overwhelmed, resulting in fewer opportunities to mitigate the cumulative burden.

Six themes, each with a set of sub-themes, were generated and have been synthesised narratively below according to the aims of the research: 1) Experiences of exposure to patient pregnancy loss, 2) perceived support available, 3) support previously used, and 4) support needed or wanted.

Illustrative quotations are provided. Within illustrative quotations the use of […] indicates part of the quotation was not presented because it was not relevant, whereas (text) indicates additional text added for clarity. Quotes are followed by participant identifier and number. To protect anonymity, we have categorised doctors, midwives and nurses as ‘clinical’, sonographers and theatre staff as ‘allied staff’, and nursing/theatre assistants and administrators as ‘support staff’.

### Experiences of exposure to patient pregnancy and neonatal loss

**Theme 1. Nobody prepared me for this:** staff across roles described exposure to pregnancy and neonatal loss as ‘stand-alone’, distinct from other clinical trauma.

1.1. Missed opportunities: Introductory training was often brief, general and insufficiently tailored to preparing staff for the emotional, and communicative challenges they might encounter. Junior medical, allied and support staff members in particular described feeling underprepared and highlighted missed opportunities during recruitment and induction processes that could be used to alert them to, and discuss, the potentially distressing nature of loss related care.

Although medical, nursing and midwifery staff described having built up resilience through their professional experience others found the lack of preparation daunting and expressed a need for improved introductory and ongoing training that began earlier in the employment process to prepare them for the specific emotional demands and communication needs of pregnancy and neonatal loss:

*“So, yeah – I do feel like maybe you should be briefed – when you’re first coming into the job (on Gynaecology ward/dealing with terminations & miscarriages)– that, this is what you can see – and the babies can be moving – and they can still be alive”* (108 Support staff)

1.2. Emotional unpreparedness: Inadequate job preparation also led many staff to report a lack of emotional preparation for loss related care, with some describing having to find their own way when encountering bereavement care for the first time:

“W*e’ve never had any training on how to, for us to cope or how to help the patients themselves manage that situation you know, and we just have to try and find our way”* (111 Allied staff)

1.3. Training disparity: *S*ome HCPs benefitted from training. However, formal preparation to manage pregnancy and neonatal loss varied both across and within staff groups with some reporting their learning was through experience only:

*“I think sometimes you just have to deal with it there and then and just if that happens and there’s no one else to deal with it, you just have to get on and do the job and then kind of process what you had to do afterwards”* (120 Clinical staff)

**Theme 2. Reaching a tipping point:** Pregnancy and neonatal loss was found to be emotionally challenging for all staff. Long, intensive shifts with little or no time at work to process their experiences and decompress, exacerbated emotional distress and reduced resilience.

2.1 Cumulative exposure: Staff described difficulty recovering between distressing cases and described emotional overload following shifts involving cumulative exposure to pregnancy and neonatal loss:

“*I had three shifts in a row where I had FMU patients (losing their babies) that affected me because it was just continuous*” (113 Clinical staff)

*“When they’ve had a morning like that where everybody has lost their baby […] it’s very difficult to switch off”* (101 Allied staff)

2.2. Personal loss: Emotional distress was further intensified for staff navigating their own pregnancy, fertility issues or having experienced their own pregnancy or neonatal loss:

*“There was a lot that was really hard to navigate, for example, being pregnant and having a visible bump and then going and looking after women or telling them that they had lost their child”* (116_Clinical staff).

*“Somebody triggered me talking about that they buried their... I wasn’t allowed to bury my baby because my baby was only 16 weeks, and so at that point it wasn’t recognised, and […] I found that really triggering. So you don’t know what’s going to trigger you if you’ve been through loss yourself and then you come back into work too quickly”* (101, Allied staff)

2.3 Resilience: Although staff described developing professional resilience to patient pregnancy and neonatal loss, nearly all described moments where the cumulative burden of exposure reached a “tipping point” where resilience felt compromised. At these times, their usual coping strategies felt insufficient, and they needed a break or extra support. Senior staff with greater experience in obstetrics, gynaecology and neonatal care, although reporting a greater sense of resilience, highlighted cumulative emotional fatigue that developed over years of repeated exposure or exposure to particularly distressing cases, such as unexpected late-gestation losses or complex fetal conditions and the impact it could have for their resilience:

*“I have days where I think God, I wonder if I can manage going in, knowing what I might be doing today”* (102 Clinical)

*“I get to the point where I take myself off and hide in the toilets… it will make me feel like I can’t be there anymore, and this job is just impossible”* (103 Support staff)

### Perception of support available

**Theme 3. Peer support versus inconsistent organisational support:** All participants identified colleagues, including immediate team members, trusted peers, and sometimes partners or friends outside of work, as their most accessible and reliable source of support.

3.1 Valued colleagues and peers: Support from peers and colleagues was valued and perceived as being readily available. This source of support was perceived as being immediately available and grounded in shared experience. Some participants reported peers and colleagues to be their only source of support:

*“I do feel well supported. I think it’s nice having a small team, so you know each other quite well and be able to talk to each other. I think that’s key in being able to talk about these things and knowing that if I needed to, my band 7 manager, if I was really struggling with a particular case or needed extra time out, I think that she – I know that would be okay”* (109 Clinical staff)

*“I think there’s very little support, if any actually, apart from each other”* (108, Allied staff)

3.2 Vague awareness of organisational support: In contrast, awareness of the range of organisational support available varied amongst staffing groups. Although some were aware that wellbeing resources within the Trust existed (e.g., “I think there’s a wellbeing page”), most reported uncertainty about what was available, how to access it, or whether it was appropriate for pregnancy and neonatal loss related distress, and few had tried to use it There was also an uncertainty about whether seeking support would involve waiting lists and limited sessions (e.g., counselling):

*“I never even knew we had a team and we had that support”* (115 Clinical staff)

“*I’d like to look into um to get more knowledge of what’s there and what you can use and things like that. Um but yeah on a day-to-day basis you wouldn’t necessarily go and look at it”* (106_Clinical staff)

3.3. Formal support exclusion: Lack of awareness of formal support was most pronounced among allied and support staff who felt “forgotten” or excluded from organisational support despite being exposed to distressing situations and bereaved families.

*“I think there’s a lot of support that we signpost or that we might be aware of for women, but I think for staff, not so much”* (109 Clinical staff)

*“I have found them (debriefs) really helpful, but I don’t know whether they’re for everyone and I’m not sure that everyone gets the same things out of them”* (110 Clinical staff) *“I feel we do get forgotten”* (111 Allied staff)

### Support previously used

**Theme 4. Reliance on informal support networks:** While theme 3 describes participants perceptions and awareness of available support, this theme focuses on the support that staff accessed and relied upon in practice. Very few staff reported using formal sources of support following exposure to patient pregnancy and neonatal loss. Instead, most relied on peers, and family.

4.1 Peer and colleague supported coping: Across departments, participants described a strong sense of camaraderie within their multi-disciplinary teams where colleagues were physically present, understood the clinical context and support from anyone in the team, from consultant to administrative staff, offered immediate validation and reassurance following exposure to loss. Staff valued these and other proactive actions (e.g., informal manager check-ins), describing them to validate their experiences, reduce feelings of isolation and self-blame and enabling time for one-to-one reflection:

*A lot of staff (come) to me, because I’m always there. We’ve got a really good team on the ward… if someone’s crying, I’ll be there to support them… We all do it for each other”* (103 Support staff)

*“I have very good friends in the specialty who I very much lean on for emotional support because they are kind of the only people that understand truly what you might experience at work”* (116 Clinical staff)

4.2 External support networks: Where peer and colleague support was not sought, often due to time pressures and limited staff availability at the end of a shift, staff reported relying on friends or partners. Experiences of this varied. While some found this useful, others noted partners and friends being unable to relate to their experiences, feeling uncomfortable disclosing the distressing nature of the experience or having no partner to confide in. Across roles, staff emphasised the importance of receiving support from individuals with either professional understanding or lived experience of pregnancy or neonatal loss:

*“I do come home and talk about kind of issues with my husband and what have you although he has no medical understanding, it’s nice to be able to do that before you go to bed, otherwise you do tend to, you know, fret about things”* (111 Allied staff)

“*Other people just don’t get it. (...). However good at listening they are (…), I just don’t think they can even understand what it’s like*” (112 Clinical staff)

4.3 Variability in support access: Some senior staff and managers described being approached with multiple concerns, including personal or mental health difficulties, sometimes involving severe distress, for which they felt ill equipped and insufficiently trained. At the same time, others recognised that some staff felt uncomfortable seeking support from managers due to fears of judgement, stigma, being seen as ‘weak’ or professionally inadequate, and a perception that their role did not legitimise their need for support:

*“We’ve reflected on this actually with colleagues about how strange it can be that you can’t talk about what we do and what we see to people and sometimes our job feels very strange”* (110 Clinical staff)

*“Because it (seeking help) feels like a weakness, doesn’t it? – you know, that they can’t manage their job”* (104 Clinical staff)

**Theme 5. Bereavement team and multi-disciplinary support buffer the stressful effects of loss:** While many participants relied primarily on informal peer support, several also described the importance of specialist bereavement services and structured multi-disciplinary support in helping staff manage the emotional impact of pregnancy and neonatal loss.

5.1 Trusted specialist support: Several staff spoke highly of the support provided by the specialist bereavement team at the Trust, whose role extended beyond direct patient care to include training, emotional support and sharing practical guidance (e.g., protocols and paperwork). Their specialist knowledge and availability made them a trusted source of both reassurance and expertise:

*“They’re (the bereavement team) available for support. So, […] if I wasn’t sure about something or if I was feeling a bit you know, sad about things, I’d be able to pick up the phone and get support from them”* (112 Clinical staff)

5.2. Valued reflections: Some staff described attending multi-disciplinary group sessions such as huddles, debriefs or more formal, structured meetings such as Perinatal Mortality and Morbidity reviews following traumatic or complex cases. Group reflective sessions that were supported or led by a psychologist were described as particularly powerful for helping staff process experiences and access further psychological support where needed. However, a few described group sessions as intimidating or uncomfortable. They were also described as inconsistent, reactive, and not available as a routine, standardised component of staff support, because they could be reliant on senior staff initiating them:

*“So, everyone was there and then everyone started saying their mind, talking about what they should have done and then what happened and then, you know, sort of like a reflection, (…) pour out our mind (..) which was very, very helpful*” (115 Support Staff)

*“’I’m not sure whether everybody would feel able to speak up”* (116, Clinical staff).

5.3: Self-initiated support: Staff who had attended specialist bereavement training described this as supportive and beneficial in enhancing their resilience. Other participants reported having sought external support (e.g., private counselling sessions) or relied on self-help methods, such as yoga exercise and journaling, to support themselves and help them process difficult or challenging experiences:

*“I reduced my hours because I was so conscious that I’d wanted to reduce the toll of what we are experiencing every day on myself”* (102 Clinical staff)

*“Doing all the other things you know like doing my yoga and being in nature”* (105 Clinical staff)

*I haven’t used any official services outside of work, but sort of I use exercise quite a lot and diet to sort of manage the way I feel* (113, Clinical staff)

### Support needed and/or wanted

**Theme 6. One size does not fit all:** Participants identified several areas where additional or improved support was needed, highlighting gaps in current provision and a desire for more accessible, consistent and tailored approaches.

6.1. Flexible, multi-modal support: Participants emphasised that one size does not fit all, describing the importance of the availability of a range of support mechanisms, such as peer support, psychologist – supported sessions, structured group debriefs or reflective practice, access to self-initiated support and informal manager check-ins. They also emphasised the need for improved specialist training and communication skills, recognising that individual needs vary across roles, workloads and clinical contexts. Participants stressed that multiple options should be available to all staff regardless of job role, level of clinical exposure or seniority:

“*Different people need (…) different things and it’s important not to do harm by sort of saying everybody’s got to do this or we’ve got to follow this process because it’s not necessarily what everybody needs or wants through it but it’s having that sort of um, the different options for people to be able to access the support they need which is tailored to the individual rather than saying it’s gotta be done this way for everybody”* (114 Clinical staff)

6.2 Timely and accessible support: Participants identified a clear need for support systems that were accessible and sensitive to the unique emotional demands associated with exposure to pregnancy and neonatal loss. There was acknowledgement that more support was needed at the time of an incident or at the end of the shift to help staff process events, including the option to take time out or use a designated wellbeing area.

*“Checking everyone’s alright at the end of the shift all it would take is for the band seven to say goodbye to every person who’s been on shift and make sure they’re okay”* (112 Clinical staff)

*“They (staff) should get the time, but it’s not always easy”* (104 Clinical staff)

6.3. Proactive, stigma free support: Staff also described the need for ongoing routine psychological support, rather than it’s provision only after severe or complex cases. Many highlighted the value of structured support such as regular check-ins, supervision, or facilitated debriefs, while others emphasised the importance of discrete, easily accessible support that could be used without stigma. Regular formal or informal check-ins (especially at the end of a difficult shift) from managers were seen as more meaningful than annual development reviews, enabling early identification of distress and creating a culture where emotional wellbeing was openly acknowledged. They also expressed a desire to be kept informed about outcomes of complex cases, especially those in allied or support roles, as this supported closure and emotional processing.

*“What I really truly believe that we need is a safe space that you have more structured debriefs with a psychologist when there has been a bad outcome”* (102 Clinical staff)

*“Often staff don’t want to be doing it (seeking support) out in a public place… I think […] access in a private setting.”* (117 Clinical staff)

6.4 Improved training and communication: Across clinical and non-clinical roles, participants consistently reported a desire for improved bereavement training on how to manage pregnancy and neonatal loss, build resilience, communicate with bereaved families, recognising cumulative stress and accessing available support. Managers similarly described a need for further training to feel confident when supporting their teams with many recognising the unpreparedness of junior staff being a problem in need of addressing. Suggested training included general wellbeing training and specific counselling courses delivered by third-sector organisations, with expertise and/or lived experience of pregnancy or neonatal loss. Participants also suggested strengthening the current provision of support from the Bereavement Team by including a nurse with specific gynaecology experience to support not only the patients experiencing pregnancy loss under 12 weeks but also for the staff who care for them:

“*I think a lot of people would benefit from more training. I think the nurses get more training… because I’m admin I don’t think I have access to as much”* (103 Support staff)

*“How do we talk to somebody who’s just lost a baby? I mean, midwives get that kind of training within their training, but we don’t you know”* (111 Support staff)

## Discussion

### Main findings

This study provides an exploration of the emotional impact of exposure to patient pregnancy and neonatal loss for clinical and non-clinical NHS staff. The findings reveal cumulative emotional burden, shaped by role, frequency of exposure, and organisational context alongside marked variability in preparedness, awareness and accessibility of support. Informal peer support emerged as the most immediate and trusted coping resource, while formal pathways were often unclear or inconsistently provided and accessed.

### Interpretation

These findings can be interpreted through established models of occupational stress and coping, which emphasise the dynamic interaction between individual resources and environmental demands [21]. Although more experienced staff in particular appeared to draw on professional resilience, distress associated with pregnancy and neonatal loss was reported across all levels of seniority. Repeated exposure, particularly in high intensity roles was associated with cumulative strain and reports of ‘tipping points’ supporting evidence that experience does not eliminate emotional burden [22]. Research indicates that cumulative exposure to distressing clinical events is a stronger predictor of psychological distress than isolated incidents, particularly when opportunity for recovery is limited [23,24]. The concept of tipping point can therefore be understood within this broader evidence base and in relation to established theories on secondary traumatic stress, compassion fatigue and vicarious trauma [25,26]. These frameworks propose that repeated exposure to others trauma, particularly in emotionally demanding clinical environments, may lead to progressive depletion of coping resources when opportunities for recovery and structured support are limited. In the present study, the reported tipping point appeared to reflect this cumulative process, whereby repeated exposure to pregnancy and neonatal loss, combined with sustained emotional labour and inconsistent access to formal support, result in a threshold beyond which the participant’s usual coping strategies were no longer sufficient. This interpretation is consistent with evidence that cumulative exposure, rather than isolated incidents is a key driver of occupational distress across healthcare settings [4,27]. Staff in specialist roles reported greater access to training and established structured support. In contrast, junior allied and support staff frequently encountered emotionally demanding situations without preparation [28]. These findings align with previous research identifying gaps in preparation for students and early career professionals [29]. Together, these findings suggest that current training models may assume that coping develops informally through experience, rather than through structured preparation and ongoing support.

A key contribution of this study is the insight into the experiences of allied and support staff. These individuals often serve as the first point of contact for distressed families yet there was a clear disparity in preparedness and access to support. This highlights how organisational systems may inadvertently overlook the needs of more junior roles and roles perceived as having less direct involvement with patients experiencing pregnancy and neonatal loss. These staff also described feeling excluded from formal reflective spaces, such as multidisciplinary debriefs, reinforcing hierarchical patterns in access to wellbeing support that have been documented elsewhere in healthcare organisations [30].

Staff emphasised the importance of timely and ongoing support, including structured check-in, and debriefs, alongside accessible and discrete support options. Informal peer support was consistently valued as an immediate buffer against distress. However, reliance on peer support may perpetuate inequity, as access varies across teams [31,32]

Training emerged as a central mechanism for improving preparedness and psychological safety across roles. Staff emphasised the need for training addressing both communication skills and emotional demands, with interdisciplinary approaches, including input from psychologists and individuals with lived experience being particularly valuable.

Taken together, these findings indicate that while informal support and individual resilience are important, reliance on personal coping is neither equitable nor sustainable. Emotional burden is cumulative, disparities in preparation persist and access to psychological support remains inconsistent. These findings therefore support the need for organisational approaches that move beyond individuals’ resilience to address structural and cultural factors [33]. The data therefore indicate a need for a co-ordinated, system wide approach that addresses training, leadership and visibility of support.

Participants described diverse preferences for support, differences in preparedness, and inconsistent access depending on role and department. These variations suggest that a standardised, top-down model is unlikely to meet the needs of all staff. Approaches grounded in codesign or coproduction would enable staff from across clinical, allied, and nonclinical roles to shape support tools, training content, and wellbeing pathways in ways that reflect the realities of their day-to-day work. While co-designed approaches are increasingly advocated in healthcare research as they offer a means of developing support tools that are responsive to the needs of diverse staff groups, the process of co-design itself presents important methodological and practical challenges. These include managing power differentials between staff groups within hierarchical clinical environments, which may include whose voices are prioritised during design discussions. These is also the potential for participation bias, whereby those with greater availability, confidence or prior engagement with research are more likely to contribute, limiting the representativeness of co-design outputs. In addition, engaging staff in co-design processes that involve reflection on emotionally distressing experiences such as pregnancy and neonatal loss may carry a risk of emotional burden and require careful facilitation and support. Translating diverse experiential accounts into actionable design outputs can also be complex, particularly when needs vary across clinical, allied, and support roles. These challenges may be mitigated through inclusive facilitation strategies, structured and psychologically safe co-design processes, and the use of multiple engagement formats (e.g., workshops, asynchronous digital input, and iterative feedback cycles) to ensure broader and more equitable participation. Moreover, despite these challenges, co-design remains a valuable approach in this context as it enables interventions to be grounded in the lived experiences of staff and responsive to variation in need across clinical, allied, and support roles. Co-designed approaches may therefore improve acceptability and sustainability of support interventions [34]. Embedding co-production in the development of wellbeing initiatives may therefore enhance both the relevance and sustainability of support mechanisms for staff exposed to pregnancy loss. It is on this basis that evidence informed recommendations are proposed in Table 1 for NHS trusts, clinical teams and organisational leadership.

**Table 1:** Evidence informed recommendations for NHS trusts, clinical teams and organisational leadership organised by level of implementation.

Immediate, local team-level actions	Theme/s	
**1. Routine wellbeing check-ins by senior staff**	4, 6	Senior staff (e.g., ward or shift managers/ senior doctors / consultants) should be encouraged to check-in with their staff who have experienced working with pregnancy loss cases during each shift. Emphasis should be placed on acknowledgment, identifying early signs of distress, promoting awareness of emotional wellbeing and adequate signposting.
**2. End of shift wellbeing checklists**	6	The implementation of a structured wellbeing checklist. Emphasis should be placed on identifying the need for immediate support.
**3. Fostering a culture of peer support**	3,6	All staff should be encouraged to check-in with each other. Emphasis should be placed on the value of informal peer support.
**4. Protected time to access support**	4,6	Enable protected time during or after shifts to access support/ debrief or have adequate time to process. Emphasis should be placed on flexibility and the ability to step away from clinical duties (even briefly) following exposure to patient pregnancy loss.
**Organisational, service-level actions**		
**1. Comprehensive assessment of staff training needs**	1, 5, 6	Routine assessments of training needs across all staff groups including new staff, students, non-clinical teams etc. Emphasis should be placed on communication skills relating to delivering bad news and supporting bereaved patients.
**2. Development of tailored training packages**	1, 6	Training should be coproduced to meet the needs of different roles and levels of experience. Emphasis should be placed on tailoring training for staff who have worked in different healthcare systems, whose first language is not English and communicating with patients.
**3. Training to enhance resilience and coping**	1, 2, 5, 6	All staff should have access to training and resources aimed at building psychological resilience. Emphasis should be placed on recognising signs of cumulative stress, strategies for emotional regulation and guidance on when and how to seek support.
**4. Increased provision and visibility of specialist psychological support**	5, 6	Trusts should support the integration of regular psychological clinics, drop-in sessions or debriefs. Emphasis should be placed on increasing the visibility and approachability of psychological services to reduce stigma associated with seeking help and encourage uptake of support.
**5. Development of dedicated wellbeing spaces for staff**	6	The provision of confidential, wellbeing spaces that are accessible to staff from any department. Emphasis should be placed on allowing respite following distressing events. It should be a pleasant and relaxing space away from clinical areas with information on support services available.
**6. Enhanced wellbeing training for managers**	6	Managers should feel they have the knowledge, tools and protocols to facilitate supporting staff adequately, e.g. targeted training in mental health awareness, trauma informed supervision and development of a specific suicide ideation protocol. Emphasis should be placed on ensuring a thorough knowledge of available support services, plus facilitating appropriate and consistent communication, support and signposting.
**Strategic, workforce development actions**		
**7. Improve recruitment processes to prepare staff**	1	Ensure recruitment materials and job description accurately outline the types of bereavement work staff may encounter and the emotional demands of the role Emphasis should be placed on discussing these expectations during interviews and guaranteeing that appropriate induction and training are provided from the outset.
**8. Include a nurse with gynaecology experience within the bereavement team**	6	Strengthen the bereavement service by incorporating a nurse with specific gynaecology expertise to support patients and staff caring for patients experiencing pregnancy loss below 12 weeks.

### Strengths and limitations

This study provides an in-depth qualitative exploration of the emotional and support needs of NHS staff exposed to pregnancy and neonatal loss across a wide range of clinical and nonclinical roles. The use of semi-structured interviews enabled participants to share rich, nuanced accounts, while the iterative approach to data collection allowed emerging issues to shape subsequent interviews and participant recruitment. Analytical rigour was strengthened through systematic coding, and reflexive team discussions. However, the sample consisted of volunteers, which may introduce bias. Those who volunteered to participate may have been more likely to have strong views or personal experiences. However, the large number of staff who expressed interest, from whom participants were purposefully sampled, suggests that such views and experiences may be widespread across the workforce rather than limited to a small group of particularly motivated individuals. Similarly, the study did not capture the experiences of staff who had changed departments or left their jobs altogether. Such, groups might have valuable perspectives on the impact of cumulative exposure or inadequate support.

Although purposive sampling was used to achieve representation across a range of clinical, allied, support, and administrative roles, participation was voluntary and recruitment was therefore dependent on staff willingness to take part. As a result, some staff groups were represented by smaller numbers of participants than others; for example, only one administrative staff member participated despite efforts to recruit across all relevant roles. The study was designed to explore shared and contrasting experiences across the workforce therefore findings relating to smaller staff groups should be interpreted with appropriate caution. However, despite smaller numbers in some roles, there was substantial consistency in the patterns of experience described across participant accounts, and no substantially new themes emerged in later interviews, suggesting sufficient depth and repetition of data to support the analytic findings.

An additional limitation was that participants were grouped into clinical, allied and support categories to protect anonymity. Consequently, it was not possible to provide a more detailed breakdown by specific professional roles. This was necessary to minimise risk of deductive disclosure, particularly given the small number of staff in some positions, but it may have limited the extent to which role specific differences in experience could be fully represented.

Moreover, as the research was conducted in a single NHS Trust that provides tertiary maternal and fetal medicine care for patients, findings may not be fully transferable to other organisational contexts. The Trust provides high-complexity maternity, fetal medicine neonatal intensive care and bereavement services. As a result, staff are more likely to encounter complex, high-acuity cases and repeated exposure to pregnancy and neonatal loss compared with staff working in smaller district general hospitals. This may have contributed to the intensity and frequency of emotional exposure described by participants, and therefore the reported experience of cumulative burden and tipping points may be more pronounced than in less specialised settings. However, several findings are likely to have relevance across other NHS settings where staff are exposed to pregnancy and neonatal loss. In particular, the cumulative emotional burden associated with repeated exposure to distressing events, reliance on informal peer support, variability in preparedness and inconsistent awareness and accessibility of formal support. Moreover, the organisational and cultural issues identified, including disparities in support access across staff groups and the need for more visible structured wellbeing support are likely to have broader relevance across NHS maternity and neonatal settings.

## Conclusion

This study highlights the significant emotional impact of pregnancy and neonatal loss on NHS staff across clinical, allied, and support roles, and the uneven access to preparation and support. While peer support was consistently valued, formal pathways were often unclear or inconsistently available. There is a need for more accessible, proactive and ongoing support, including routine psychological input, clearer signposting, and dedicated time for support. Crucially, the findings indicate that future support initiatives should be co-designed with staff to ensure it meets the needs of those exposed to pregnancy and neonatal loss in their day-to-day roles. By broadening understanding beyond traditionally studied clinical groups, this study offers actionable insights for developing support systems that are inclusive, responsive, and sustainable within NHS settings.

## Supporting information

S1 Table Type of loss and frequency of exposure across hospital departments. (DOCX)

S1 Checklist Standards for Reporting Qualitative Research (SRQR). (DOCX)

## References

[1] SANDS. Miscarriage, stillbirth and neonatal death. https://www.sands.org.uk/about/miscarriage-stillbirth-and-neonatal-death. 2020.

[2] Brier N. Grief following miscarriage: a comprehensive review of the literature. J Womens Health (Larchmt). 2008;17(3):451–64. doi: 10.1089/jwh.2007.0505 18345996

[3] Cuenca D. Pregnancy loss: consequences for mental health. Front Glob Womens Health. 2023;3:1032212. doi: 10.3389/fgwh.2022.1032212 36817872 PMC9937061

[4] Gold KJ, Kuznia AL, Hayward RA. How physicians cope with stillbirth or neonatal death: a national survey of obstetricians. Obstet Gynecol. 2008;112(1):29–34. doi: 10.1097/AOG.0b013e31817d0582 18591304

[5] Gold KJ, Sen A, Hayward RA. Marriage and cohabitation outcomes after pregnancy loss. Pediatrics. 2010;125(5):e1202-7. doi: 10.1542/peds.2009-3081 20368319 PMC2883880

[6] Kemp H. The Second Victims: A Grounded Theory Explanation of the Experience and Impact of Traumatic Births Amongst Midwives and Obstetricians. UCL (University College London). 2020.

[7] Pezaro S, Clyne W, Turner A, Fulton EA, Gerada C. “Midwives Overboard!” Inside their hearts are breaking, their makeup may be flaking but their smile still stays on. Women Birth. 2016;29(3):e59-66. doi: 10.1016/j.wombi.2015.10.006 26522961

[8] Brückner TY, Heemelaar S, Endjala T, van den Akker T. Healthcare worker burnout: exploring the experiences of doctors working in a maternity unit in Namibia. BMC Health Serv Res. 2024;24(1):362. doi: 10.1186/s12913-024-10845-z 38515163 PMC10958874

[9] Butcher I, Morrison R, Balogun O, Duncan H, St Louis K, Webb S, et al. Burnout and coping strategies in pediatric and neonatal intensive care staff. Clinical Practice in Pediatric Psychology. 2024;12(1):16–30. doi: 10.1037/cpp0000474

[10] Hunter B, Fenwick J, Sidebotham M, Henley J. Midwives in the United Kingdom: levels of burnout, depression, anxiety and stress and associated predictors. Midwifery. 2019;79:102526. doi: 10.1016/j.midw.2019.08.008 31473405

[11] Aydın R, Aktaş S. Midwives’ experiences of traumatic births: a systematic review and meta-synthesis. Eur J Midwifery. 2021;5:31. doi: 10.18332/ejm/138197 34386725 PMC8312097

[12] Gandino G, Bernaudo A, Di Fini G, Vanni I, Veglia F. Healthcare professionals’ experiences of perinatal loss: a systematic review. J Health Psychol. 2019;24(1):65–78. doi: 10.1177/1359105317705981 28810447

[13] Sheen K, Spiby H, Slade P. Exposure to traumatic perinatal experiences and posttraumatic stress symptoms in midwives: prevalence and association with burnout. Int J Nurs Stud. 2015;52(2):578–87. doi: 10.1016/j.ijnurstu.2014.11.006 25561076

[14] McCreight BS. Perinatal loss: a qualitative study in Northern Ireland. Omega (Westport). 2008;57(1):1–19. doi: 10.2190/OM.57.1.a 18507324

[15] NHS survey. NHS Staff Survey Results 2021. 2021. https://www.nhsemployers.org/articles/nhs-staff-survey-2021-results

[16] BNSSG Healthier Together. All Age Mental Health and Wellbeing Strategy. 2024. https://bnssghealthiertogether.org.uk/wp-content/uploads/2024/05/BNSSG-All-Age-Mental-health-and-Wellbeing-Strategy_final.pdf

[17] Royal Collegde of Midwives. Caring for You Campaign Report. 2020. https://rcm.org.uk/caring-for-you/

[18] Wallbank S, Robertson N. Predictors of staff distress in response to professionally experienced miscarriage, stillbirth and neonatal loss: a questionnaire survey. Int J Nurs Stud. 2013;50(8):1090–7. doi: 10.1016/j.ijnurstu.2012.11.022 23312617

[19] Kelly RM. The Effects of Debriefing on Nurse Distress after Perinatal Loss. 2021.

[20] Braun V, Clarke V. Reflecting on reflexive thematic analysis. Qualitative Research in Sport, Exercise and Health. 2019;11(4):589–97. doi: 10.1080/2159676x.2019.1628806

[21] Biggs A, Brough P, Drummond S. Lazarus and Folkman’s psychological stress and coping theory. The handbook of stress and health: A guide to research and practice. 2017. 349–64.

[22] Nuzum D, Meaney S, O’Donoghue K. The impact of stillbirth on consultant obstetrician gynaecologists: a qualitative study. BJOG. 2014;121(8):1020–8. doi: 10.1111/1471-0528.12695 24589177

[23] Papadatou D. A Proposed model of health professionals’ grieving process. Omega (Westport). 2000;41(1):59–77. doi: 10.2190/tv6m-8yna-5dyw-3c1e

[24] Cocker F, Joss N. Compassion fatigue among healthcare, emergency and community service workers: a systematic review. Int J Environ Res Public Health. 2016;13(6):618. doi: 10.3390/ijerph13060618 27338436 PMC4924075

[25] McCann IL, Pearlman LA. Vicarious traumatization: a contextual model for understanding the effects of trauma on helpers. Journal of Traumatic Stress. 1990;3(1):131–49.

[26] Figley CR. Compassion fatigue: Coping with secondary traumatic stress disorder in those who treat the traumatized. Routledge. 2013.

[27] Beadle ES, Walecka A, Sangam AV, Moorhouse J, Winter M, Munro Wild H, et al. Triggers and factors associated with moral distress and moral injury in health and social care workers: a systematic review of qualitative studies. PLoS One. 2024;19(6):e0303013. doi: 10.1371/journal.pone.0303013 38935754 PMC11210881

[28] Fenwick J, Jennings B, Downie J, Butt J, Okanaga M. Providing perinatal loss care: satisfying and dissatisfying aspects for midwives. Women Birth. 2007;20(4):153–60. doi: 10.1016/j.wombi.2007.09.002 17964235

[29] Legere LE, Wallace K, Bowen A, McQueen K, Montgomery P, Evans M. Approaches to health-care provider education and professional development in perinatal depression: a systematic review. BMC Pregnancy Childbirth. 2017;17(1):239. doi: 10.1186/s12884-017-1431-4 28738855 PMC5525243

[30] Essex R, Kennedy J, Miller D, Jameson J. A scoping review exploring the impact and negotiation of hierarchy in healthcare organisations. Nurs Inq. 2023;30(4):e12571. doi: 10.1111/nin.12571 37338510

[31] Habib M, Palachi A, Korman MB, Steinberg R, Cocco C, Martin-Doto C. Patterns of distress and supportive resource use by healthcare workers during the COVID-19 pandemic. MDPI. 2025. 10.3390/healthcare13151785 PMC12346357 40805818

[32] Lee BEC, Ling M, Boyd L, Olsson CA, Harvey HML, Sheen J. Three years of pandemic stress and staffing challenges: a retrospective qualitative study of COVID-19 impacts on frontline healthcare workers’ mental health and wellbeing. BMC Psychiatry. 2025;25(1):1036. doi: 10.1186/s12888-025-07348-4 41168749 PMC12573931

[33] Maben J, Taylor C, Jagosh J, Carrieri D, Briscoe S, Klepacz N, et al. Causes and solutions to workplace psychological ill-health for nurses, midwives and paramedics: the Care Under Pressure 2 realist review. 2024. 10.3310/TWDU4109 38662367

[34] Grindell C, Coates E, Croot L, O’Cathain A. The use of co-production, co-design and co-creation to mobilise knowledge in the management of health conditions: a systematic review. BMC Health Serv Res. 2022;22(1):877. doi: 10.1186/s12913-022-08079-y 35799251 PMC9264579

