# Supplementary material for: Examining the mental health support needs of NHS staff exposed to patient pregnancy and neonatal loss: A qualitative study
Source: PLoS One. 2026 Sep 17;21(9):e0358321. doi: 10.1371/journal.pone.0358321 (PMC13585218; doi:10.1371/journal.pone.0358321)
Supplement: Supplementary file 1 [file pone.0358321.s001.docx]

Supplementary Table 1. Type of loss and frequency of exposure across hospital departments.

| **Department** | **Type of pregnancy loss** |
| --- | --- |
| Sonography | Loss from very early (i.e., before 8 weeks) to late pregnancy.  Daily exposure to pregnancy loss and fetal abnormalities. Sonographers can be the first to break bad news to parents |
| Early Pregnancy Clinic & Gynaecology ward | Loss between 6 and 19+6 weeks,  Daily exposure to early pregnancy loss |
| Fetal Medicine Unit | Loss or discussions regarding severe fetal anomaly and the option of termination of pregnancy at any time in the pregnancy  Weekly exposure to complex pregnancy loss & fetal abnormalities leading to termination of pregnancy and occasionally feticide |
| Delivery Suite | Losses above 20 weeks gestation, including late miscarriages, stillbirths, early neonatal deaths and termination of pregnancy for fetal or maternal reasons.  Approximately 2 a month exposure. |
| Operating Theatre | Losses at all gestations including late miscarriages, stillbirths, early neonatal deaths and termination of pregnancy for fetal or maternal reasons, who have required a delivery in the maternity theatres. Loss of a baby can be the result of obstetric emergencies when surgery has been the last hope.  Weekly exposure. |
| Neonatal Intensive Care Unit (NICU) | Early neonatal death either on the delivery suite following a resuscitation situation or after babies are transferred to NICU.  Approximately 2 a month exposure. |
