# Supplementary material for: Examining the mental health support needs of NHS staff exposed to patient pregnancy and neonatal loss: A qualitative study
Source: PLoS One. 2026 Sep 17;21(9):e0358321. doi: 10.1371/journal.pone.0358321 (PMC13585218; doi:10.1371/journal.pone.0358321)
Supplement: Supplementary file 3 [file pone.0358321.s004.docx]

We thank the editors and reviewers for their constructive and detailed feedback. We have revised the manuscript in response to all comments. All changes are marked in the tracked version. Line numbers refer to the manuscript with tracked changes.

**Reviewer #1: MAJOR COMMENTS
1. Methodological Framework and Analytical Rigour
The authors state thematic analysis (Braun & Clarke) was used, but describe an approach that is inconsistent with a fully reflexive stance. Themes are explicitly "mapped to the study aims," which reflects a more framework-driven or deductive approach. This tension is not acknowledged or justified. The authors should either: (a) transparently describe their approach as a hybrid or directed thematic analysis, or (b) explain how inductive theme generation was reconciled with aim-mapping without compromising analytical integrity.**

REPLY: Thank you to the reviewer for this important observation. We agree that our original wording did not sufficiently distinguish between the inductive development of themes and the later stage of relating findings back to the study aims. We have revised the Methods section to clarify that the analysis followed an iterative and reflexive thematic analysis approach, with themes generated inductively from the data. We have highted that our approach deviates from Braun and Clarke’s as the themes have been interpreted in relation to the research aims during the write-up phase and presented in such a way to aid future intervention development. We have also clarified the collaborative and reflexive nature of the analytic process. The methods section now states:

*“Interviews were audio recorded and transcribed verbatim by a professional transcription service. NVivo software (QSR International Pty Ltd.,) was used to manage and analyse data using thematic analysis (20) following an iterative, interpretive, and reflexive approach. Transcripts were read several times for familiarisation, and initial codes were generated inductively by DJ to capture meaningful features of the data. CH independently read and coded a substantial proportion of the transcripts, contributing alternative interpretations, supporting reflexivity and the development of a richer, more nuanced analysis. Throughout the analysis, DJ and CH regularly met with SN (Consultant Obstetrician and Subspecialist in Fetal/Maternal Medicine), MH (Lead Neonatal Nurse for Bereavement), HF (Psychologist and Behavioural Scientist with expertise in qualitative research) to reflect on assumptions and discuss analytic decisions while considering how differing perspectives and backgrounds may have shaped interpretation of the data. Sub-themes were developed iteratively through close engagement with the dataset and were generated from patterns identified across participant accounts rather than being predetermined by the study aims. Subthemes were further refined and organised into broader overarching themes. Deviating from Braun & Clarke’s approach to reporting findings, the themes were organised in relation to the aims of the research to support coherent presentation and interpretation of the findings which are intended to inform future intervention development”* Line 129-148.

**2. Insufficient Reporting of Reflexivity
Reflexivity is poorly addressed. The study involves a single interviewer (DJ) who is also a researcher embedded in the institutional setting. No positionality statement is provided, and there is no discussion of how the team's professional backgrounds, institutional affiliations, or personal experiences may have shaped data collection and analysis. This is a fundamental requirement for qualitative research and must be added.**

REPLY: Thank you for highlighting the need for greater reflexive transparency. We agree that the original manuscript did not provide sufficient detail regarding the researcher’s positionality. We have now added a reflexive statement in the methos section describing DJs role in addition to highlighting consideration given to the potential influence of her positioning on participant responses and interview dynamics. In the methods section it now states:

*“Interviews were carried out online (MS Teams) by DJ (a research associate with a background in health research including topics related to pregnancy, breastfeeding and perinatal mental health in addition to previous midwifery experience). A flexible topic guide, informed by the aims of the study, was developed and adapted appropriately for the participant’s job role. Reflexive consideration was given to how DJs positioning may have influenced participant responses and interview dynamics”.* Lines 122-128

We have also added positioning for the other researchers involved in the analysis:

*“CH (a behavioural scientist with expertise in sexual and reproductive health) independently read and coded a substantial proportion of the transcripts, contributing alternative interpretations, supporting reflexivity and the development of a richer, more nuanced analysis. Throughout the analysis, DJ and CH regularly met with SN (Consultant Obstetrician and Subspecialist in Fetal/Maternal Medicine), MH (Lead Neonatal Nurse for Bereavement), HF (Psychologist and Behavioural Scientist with expertise in qualitative research) to reflect on assumptions and discuss analytic decisions while considering how differing perspectives and backgrounds may have shaped interpretation of the data. Sub-themes were developed iteratively through close engagement with the dataset and were generated from patterns identified across participant accounts rather than being predetermined by the study aims. Subthemes were further refined and organised into broader overarching themes. Deviating from Braun & Clarke’s approach to reporting findings the themes were organised in relation to the aims of the research to support coherent presentation and interpretation of the findings which are intended to inform future intervention development”.* Lines 134-148

**3. Saturation and Sample Justification
The authors recruited 20 of 49 volunteers via purposive sampling but provide no rationale for why 20 participants were sufficient. There is no discussion of data saturation or information power. Given the heterogeneous sample (seven departments, three staff categories, varied seniority), the adequacy of the sample for capturing the full range of experiences warrants explicit justification.**

REPLY: Thank you. We thank the reviewer for this important observation. The study aimed to capture a breadth of perspectives across staff groups exposed to pregnancy and neonatal loss rather than to achieve thematic saturation within each individual professional subgroup. Whilst we acknowledge we deviated from Braun & Clarke’s approach to reporting themes; we maintained commitment to their approach through the rejection of the concept of saturation which they position as a positivist approach to qualitative research (please see: Braun & Clarke (2021) [https://doi.org/10.1080/2159676X.2019.1704846\](https://doi.org/10.1080/2159676X.2019.1704846%22%20)). Recruitment was voluntary, and although purposive sampling was used to achieve representation across clinical, allied, support, and administrative roles, participation was dependent on staff expressing interest in taking part. As a result, some groups were represented by smaller numbers of participants, including administrative staff, where only one staff member volunteered to participate.

The sample size was also shaped by the resources available to support recruitment, data collection, transcription, and analysis. Given the exploratory qualitative design, the intention was not to generate profession-specific findings, but rather to identify shared and contrasting experiences across a range of staff groups exposed to pregnancy and neonatal loss within the organisation.

We have added the following to the strengths and limitations section, to acknowledge this potential limitation:

*“Although purposive sampling was used to achieve representation across a range of clinical, allied, support, and administrative roles, participation was voluntary and recruitment was therefore dependent on staff willingness to take part. As a result, some staff groups were represented by smaller numbers of participants than others; for example, only one administrative staff member participated despite efforts to recruit across all relevant roles. The study was designed to explore shared and contrasting experiences across the workforce therefore findings relating to smaller staff groups should be interpreted with appropriate caution. However, despite smaller numbers in some roles, there was substantial consistency in the patterns of experience described across participant accounts, and no substantially new themes emerged in later interviews, suggesting sufficient depth and repetition of data to support the analytic findings”* Lines 551-561

**4. Consent Reporting Inconsistency
The methods section states participants provided "verbal informed consent," while the ethics statement to the journal says "informed consent" without specifying the form. For a study involving sensitive topics and audio recording, verbal-only consent requires explicit justification. If written consent was also obtained, this should be clarified. PLOS ONE requires precision on this point.**

REPLY: Apologies for this oversight, we have now specified in the ethics statement for the journal that verbal informed consent was obtained. We now have the following included in the participants and sampling section of the manuscript as per journal requirements highlighted above:

*“Given the remote interview format participants were asked to provided verbal informed consent prior to participation, which was approved by the National Health Service, Health Research Authority, Research Ethics Committee (25/HRA/2520 IRAS: 357165) and the University of Bristol Faculty of Health Sciences Research Ethics Committee (FREC) (REF: 25063), Participants were sent a participant information sheet in advance of the interview and had the opportunity to ask questions before consent was obtained. Verbal consent was obtained by DJ at the start of each interview and was audio-recorded as part of the interview recording, thereby providing a documented record of consent. Consent recordings were stored securely on encrypted institutional servers, separately from anonymised interview data. Participants were reimbursed with a shopping voucher as a thank you for their time.”* Line 111-121

**5. Reference Error — Reference 8
Reference 8, cited to support emotional distress and burnout in O&G staff, is incorrectly cited. The reference listed is Lisy et al. (2018) on "lesbian, gay, and bisexual people with cancer care" — entirely unrelated to the claim it supports. This must be corrected with an appropriate citation.**

REPLY: Apologies for this error. We have now replaced the reference.

**6. Data Availability Statement
PLOS ONE requires that data underlying findings be made "fully available and without restriction" unless a specific exemption applies. The statement that data are "available from the corresponding author upon reasonable request" does not meet this requirement. The authors must deposit anonymised data in an appropriate repository or provide a detailed justification for restriction (e.g., participant confidentiality, ethics committee prohibition).**
REPLY: We thank the reviewer for highlighting the journal’s data availability requirements. We recognise the importance of transparency and open data principles. However, in this study full anonymisation of the qualitative dataset is not sufficient to guarantee participant confidentiality. In line with PLOS ONE data sharing policies, we have revised the Data Availability Statement to reflect this:

*“The dataset comprises in-depth interview transcripts from a single NHS organisation with a small and identifiable workforce, meaning that even anonymised data carry a risk of deductive disclosure. Therefore, full data sharing is restricted. Requests for access to anonymised excerpts may be considered on reasonable request, subject to appropriate governance approvals. Data access requests should be directed to the University of Bristol Research Governance Team (research-governance@bristol.ac.uk), which is the study sponsor. Requests will be considered in line with applicable ethical approvals, participant consent, and institutional data governance procedures.”* Line 595-603

**MINOR COMMENTS
7. Abstract Formatting
Theme (1) in the Results ("nobody prepared me for this") is not capitalised, inconsistent with the other themes. Capitalisation should be standardised throughout the abstract and manuscript.**

REPLY: Many thanks. We have amended so all themes are not capitalised.

**8. Typographical and Grammatical Errors
Several minor errors require correction: (a) "its'" (p. 15) should be "its"; (b) a comma splice appears in the Conclusion ("support., including"); (c) "Bristol Bristol" appears twice in the affiliations list and should be corrected; (d) missing comma after aim (3) in the Introduction ("3) support previously used 4) support needed or wanted").**

REPLY: Thank you for highlighting these errors. They have now been addressed.

**9. Theme Overlap Between Themes 3 and 4
Themes 3 (peer support vs. organisational support) and 4 (reliance on informal support networks) are conceptually overlapping. The distinction between "perception of support available" and "support previously used" as organising frames is understandable, but the narrative presentation blurs this boundary. The authors should sharpen the conceptual distinction and ensure each theme presents genuinely distinct analytical content.**

REPLY: REPLY: Thank you. We have revised the manuscript in two ways to improve conceptual clarity. Firstly, we have retained both themes but clarified their distinct focus by adding the following statement at the beginning of theme 4: *“While theme 3 describes participants perceptions and awareness of available support, this theme focuses on the support that staff actually accessed and relied upon in practice”.* Second, we have refined the thematic structure by introducing subthemes as suggested by reviewer 1. Together we feel these revisions improve clarity.

**10. Table 2 — Recommendations
The 13 recommendations in Table 2 are useful practically but are largely descriptive and not sufficiently grounded in the data. Each recommendation should be explicitly linked to a specific finding or theme. Additionally, the label "Gold standard" is not justified by the evidence base from a single-site qualitative study and should be revised to something more appropriately qualified (e.g., "Evidence-informed recommendations").**

REPLY: Thank you. We have changed this to “Evidence-informed recommendations” as recommended.

We have also added a column to show the theme/s from which the recommendations derive and structured Table 2 in line with suggestions from reviewer 2.

**11. Transferability
The authors acknowledge single-site limitations but do not sufficiently discuss transferability. A brief reflection on which findings are likely context-specific to tertiary, high-complexity care settings versus those that may generalise to other NHS trusts would strengthen the discussion.**

REPLY: Thank you for this helpful suggestion. We have expanded the strengths and limitations section to provide greater consideration of transferability. We have included:

*“The Trust provides high-complexity maternity, fetal medicine neonatal intensive care and bereavement services. As a result, staff are more likely to encounter complex, high-acuity cases and repeated exposure to pregnancy and neonatal loss compared with staff working in smaller district general hospitals. This may have contributed to the intensity and frequency of emotional exposure described by participants, and therefore the reported experience of cumulative burden and tipping points may be more pronounced than in less specialised settings. However, several findings are likely to have relevance across other NHS settings where staff are exposed to pregnancy and neonatal loss. In particular, the cumulative emotional burden associated with repeated exposure to distressing events, reliance on informal peer support, variability in preparedness and inconsistent awareness and accessibility of formal support. Moreover, the organisational and cultural issues identified, including disparities in support access across staff groups and the need for more visible structured wellbeing support are likely to have broader relevance across NHS maternity and neonatal settings.”* Line 570-583

**12. Missing Reporting Checklist**
**The manuscript does not appear to reference or append a qualitative reporting checklist (e.g., COREQ or SRQR). PLOS ONE strongly encourages the use of reporting guidelines for qualitative studies. Authors should complete and submit the relevant checklist.**
REPLY: Thank you. We have now included a SRQR checklist with the submission.

**ADDITIONAL COMMENTS ON PRESENTATION AND RESULTS REPORTING
13. Absence of Subthemes
Six broad themes are presented, but none are developed with subthemes despite the richness and heterogeneity of the data evident in the quotes. For example, Theme 2 ("Reaching a tipping point") encompasses at least three distinct experiential dimensions — cumulative shift-based fatigue, personal fertility/loss history compounding professional distress, and seniority-related differences in resilience — yet these are collapsed into a single undifferentiated theme narrative. Similarly, Theme 6 ("One size does not fit all") bundles training needs, structural support preferences, timing of support, and privacy concerns without analytical distinction. The absence of subthemes limits the depth and precision of the analysis and understates the complexity of participants' experiences. The authors should consider developing an explicit thematic hierarchy with clearly labelled subthemes under each main theme, accompanied by brief descriptions of how subthemes relate to the overarching theme.**

REPLY: Thank you for this helpful suggestion. We have revised the Results section to introduce a clearer thematic hierarchy. Each of the six overarching themes now includes explicitly labelled subthemes that reflect distinct dimensions of participants’ experiences. This refinement improves analytical transparency, better reflects the heterogeneity of the dataset, and strengthens the interpretive depth of the analysis while preserving the coherence of the overarching themes.

**14. Illustrative Quotes Sequestered in a Table Rather Than Integrated into the Narrative
The decision to present all illustrative quotations in a standalone table (Table 1) rather than embedding them within the thematic narrative is a significant presentational weakness with analytical consequences. In qualitative research, quotes serve not merely as illustrations but as the evidential foundation for interpretive claims. Separating them from the narrative text prevents readers from evaluating the interpretive logic — that is, from judging whether the authors' analytical claims are warranted by the data. As presented, readers must cross-reference between the narrative and the table, disrupting the reading flow and obscuring the relationship between data and interpretation. This is not standard practice in qualitative thematic analysis reporting. The authors should embed relevant quotes directly within the thematic narrative at the point where the interpretive claim is made, following the established convention of: claim → quote → interpretive commentary. A supplementary table may be retained if desired, but it cannot substitute for in-text integration of evidence.**

REPLY: Thank you. We have deleted Table 1 and integrated the quotes into the manuscript as suggested and feel it strengthens the presentation and flow of the results.

**Reviewer #2: This review examines the manuscript titled “Examining the mental health support needs of NHS staff exposed to patient pregnancy and neonatal loss: a qualitative study” (PONE-D-26-16902).
The paper addresses a gap in the literature by including allied and non-clinical staff, who are often overlooked in research concerning perinatal loss.
The study is well-grounded and methodologically sound. However, the following areas require further attention:

1. Abstract
Line 32: The abstract lists “Experiences of exposure,” “perceived support,” etc., as “Main outcome measures”. In qualitative research, these are typically referred to as research questions or study aims rather than “outcome measures,” which is a term more suited to quantitative or clinical trials.**

REPLY: Thank you. We have changed this to be study aims and moved this section to above the methods section of the abstract. The abstract now states:

*“Study aims: To explore staff experiences of exposure to pregnancy and neonatal loss, perceptions of available support, support previously used, and needed or wanted”*

**Line 44: The conclusion in the abstract is somewhat generic. It would benefit from highlighting the specific “tipping point” concept or the unique vulnerability of allied/support staff identified in the results.**
REPLY: Thank you for highlighting this important omission. We have added:

*“Findings highlight a “tipping point” where cumulative exposure to pregnancy and neonatal loss can overwhelm coping capacity, particularly among staff in junior, allied and support roles who often report lower levels of preparation and access to formal support”.*

**2. Methodology
Sampling
• The authors recruited 20 participants across a wide array of roles: doctors, nurses, nursing assistants, theatre staff, sonographers, administrators, and midwives. While purposive sampling was used, it is unclear if thematic saturation was reached for each of these distinct groups. For example, only one administrative staff member was interviewed. The authors should clarify whether the findings for these smaller subgroups are sufficiently robust or should be presented as preliminary.**

REPLY: We thank the reviewer for this important observation. As stated above in response to reviewer 1, the study aimed to capture a breadth of perspectives across staff groups exposed to pregnancy and neonatal loss rather than to achieve thematic saturation within each individual professional subgroup. Whilst we acknowledge we deviated from Braun & Clarke’s approach to reporting themes; we maintained commitment to their approach through the rejection of the concept of saturation which they position as a positivist approach to qualitative research (please see: Braun & Clarke (2021) [https://doi.org/10.1080/2159676X.2019.1704846\](https://doi.org/10.1080/2159676X.2019.1704846%22%20)). Recruitment was voluntary, and although purposive sampling was used to achieve representation across clinical, allied, support, and administrative roles, participation was dependent on staff expressing interest in taking part. As a result, some groups were represented by smaller numbers of participants, including administrative staff, where only one staff member volunteered to participate.

The sample size was also shaped by the resources available to support recruitment, data collection, transcription, and analysis. Given the exploratory qualitative design, the intention was not to generate profession-specific findings, but rather to identify shared and contrasting experiences across a range of staff groups exposed to pregnancy and neonatal loss within the organisation.

We have added the following to the strengths and limitations section to acknowledge this potential limitation:

*“Although purposive sampling was used to achieve representation across a range of clinical, allied, support, and administrative roles, participation was voluntary and recruitment was therefore dependent on staff willingness to take part. As a result, some staff groups were represented by smaller numbers of participants than others; for example, only one administrative staff member participated despite efforts to recruit across all relevant roles. The study was designed to explore shared and contrasting experiences across the workforce therefore findings relating to smaller staff groups should be interpreted with appropriate caution. However, despite smaller numbers in some roles, there was substantial consistency in the patterns of experience described across participant accounts, and no substantially new themes emerged in later interviews, suggesting sufficient depth and repetition of data to support the analytic findings”* Line 551-561

**• The manuscript mentions an “iterative approach” to the topic guide and sampling. Providing a brief example of how the topic guide evolved based on early interviews would strengthen the “Methods” section and demonstrate analytical rigour.**
REPLY: Thank you. We have added the following to the Methods section:

*“As data collection progressed, emerging findings also informed decisions about recruitment, with additional staff groups identified as important to include in order to further explore developing themes and ensure adequate representation across relevant roles. For example, later interviews prompted further sampling of allied staff and refinement of interview prompts to explore areas such as preparedness and access to informal support in greater depth”* Line 105-110

**Consent Procedures
• The text notes that participants provided verbal informed consent (line 100). The authors should explicitly state if this was approved by the Ethics Committee instead of written consent, and how this verbal consent was recorded or witnessed to meet standard ethical reporting guidelines.**

REPLY: Thank you. In line with the requirements of the journal (highlighted above) we have changed this to state:

*“Given the remote interview format participants were asked to provided verbal informed consent prior to participation, which was approved by the National Health Service, Health Research Authority, Research Ethics Committee (25/HRA/2520 IRAS: 357165) and the University of Bristol Faculty of Health Sciences Research Ethics Committee (FREC) (REF: 25063), Participants were sent a participant information sheet in advance of the interview and had the opportunity to ask questions before consent was obtained. Verbal consent was obtained by DJ at the start of each interview and was audio-recorded as part of the interview recording, thereby providing a documented record of consent. Consent recordings were stored securely on encrypted institutional servers, separately from anonymised interview data. Participants were reimbursed with a shopping voucher as a thank you for their time.”* Line 111-121

**3. Results
• There is significant conceptual overlap between Theme 3 (Peer support versus inconsistent organisational support) and Theme 4 (Reliance on informal support networks). Theme 4 essentially explores the usage of what Theme 3 identifies as available. The authors should consider merging these or more clearly distinguishing the “perception” of support from the “behavioural reliance” on it.**

REPLY: Thank you. As above in response to reviewer 1 we have revised the manuscript in two ways to improve conceptual clarity. First, we have retained both themes but clarified their distinct focus by adding the following statement at the beginning of theme 4: *“While theme 3 describes participants perceptions and awareness of available support, this theme focuses on the support that staff actually accessed and relied upon in practice”.* Second, we have refined the thematic structure by introducing subthemes as suggested by reviewer 1. Together we feel these revisions improve clarity.

**• To protect anonymity, roles were grouped into ‘clinical’, ‘allied’, and ‘support’. However, this occasionally obscures important context. For instance, the “feticide” mentioned as a stressor is likely specific to a few medical roles. The authors should ensure that by grouping, they don’t lose the nuance of how specific clinical tasks impact mental health.**
REPLY: We thank the reviewer for this important observation regarding the potential loss of nuance arising from grouping participants into broad occupational categories. We agree that this may obscure some role-specific differences in experience. However, this categorisation was necessary to protect participant anonymity. We have now clarified this in the Limitations section:

*“An additional limitation was that participants were grouped into clinical, allied and support categories to protect anonymity and it was not possible to provide a more detailed breakdown by specific professional roles. This was necessary to minimise risk of deductive disclosure, particularly given the small number of staff in some positions, but it may have limited the extent to which role specific differences in experience could be fully represented”* Line 562-567

**4. Discussion
• The concept of a “tipping point” is a major finding. The discussion would be strengthened by linking this more clearly to existing theories of secondary traumatic stress or compassion fatigue, which are mentioned in the introduction but are less integrated into the interpretation of the “tipping point”.**

REPLY: Thank you for his helpful suggestion. We have expanded the discussion to include:

“The concept of tipping point can therefore be understood within this broader evidence base and in relation to established theories on secondary traumatic stress, compassion fatigue and vicarious trauma (25, 26). These frameworks propose that repeated exposure to others trauma, particularly in emotionally demanding clinical environments, may lead to progressive depletion of coping resources when opportunities for recovery and structured support are limited. In the present study, the reported tipping point appeared to reflect this cumulative process, whereby repeated exposure to pregnancy and neonatal loss, combined with sustained emotional labour and inconsistent access to formal support, result in a threshold beyond which the participant’s usual coping strategies were no longer sufficient. This interpretation is consistent with evidence that cumulative exposure, rather than isolated incidents is a key driver of occupational distress across healthcare settings (4, 27)”. Line 459-470

**• The study was conducted in a single NHS Trust that provides tertiary care. The authors correctly identify this as a limitation, but they should further discuss how the “high-risk, complex” nature of this specific trust might have skewed the results compared to a smaller community-based maternity unit.**
REPLY: Thank you. In line with your suggestions and reviewer 1 we have added the following in the strengths and limitations section:

*“The Trust provides high-complexity maternity, fetal medicine neonatal intensive care and bereavement services. As a result, staff are more likely to encounter complex, high-acuity cases and repeated exposure to pregnancy and neonatal loss compared with staff working in smaller district general hospitals. This may have contributed to the intensity and frequency of emotional exposure described by participants, and therefore the reported experience of cumulative burden and tipping points may be more pronounced than in less specialised settings. However, several findings are likely to have relevance across other NHS settings where staff are exposed to pregnancy and neonatal loss. In particular, the cumulative emotional burden associated with repeated exposure to distressing events, reliance on informal peer support, variability in preparedness and inconsistent awareness and accessibility of formal support. Moreover, the organisational and cultural issues identified, including disparities in support access across staff groups and the need for more visible structured wellbeing support are likely to have broader relevance across NHS maternity and neonatal settings.”* Line 570-583

**5. Recommendations (Table 2)
• Table 2 provides 13 “gold standard” recommendations. While comprehensive, this list is quite long for a healthcare lead to implement. The authors should consider categorising these (e.g., Immediate vs. Long-term, or Local vs. Organisational) or identifying the “top three” most critical interventions based on participant feedback.**

REPLY: Thank you for this helpful suggestion. We have reorganised Table 2 to improve usability for clinical and managerial audiences. Rather than reducing the number of recommendations, we have structured them into three implementation levels: immediate team-based actions, organisational/service-level interventions, and strategic workforce development initiatives. This categorisation aims to support clearer prioritisation and facilitate implementation within healthcare settings with varying resource constraints.

**• The study advocates for co-designed support tools. It would be helpful to briefly discuss the practical barriers to co-design in a high-pressure NHS environment (e.g., staff time and funding) and how they might be mitigated.**
REPLY: Thank you for this helpful suggestion. We have added:

*“While co-designed approaches are increasingly advocated in healthcare research as they offer a means of developing support tools that are responsive to the needs of diverse staff groups, the process of co-design itself presents important methodological and practical challenges. These include managing power differentials between staff groups within hierarchical clinical environments, which may include whose voices are prioritised during design discussions. These is also the potential for participation bias, whereby those with greater availability, confidence or prior engagement with research are more likely to contribute, limiting the representativeness of co-design outputs. In addition, engaging staff in co-design processes that involve reflection on emotionally distressing experiences such as pregnancy and neonatal loss may carry a risk of emotional burden and require careful facilitation and support. Translating diverse experiential accounts into actionable design outputs can also be complex, particularly when needs vary across clinical, allied, and support roles. These challenges may be mitigated through inclusive facilitation strategies, structured and psychologically safe co-design processes, and the use of multiple engagement formats (e.g., workshops, asynchronous digital input, and iterative feedback cycles) to ensure broader and more equitable participation. Moreover, despite these challenges, co-design remains a valuable approach in this context as it enables interventions to be grounded in the lived experiences of staff and responsive to variation in need across clinical, allied, and support”* Line 506-524

**6. Minor/Technical Issues
• The current statement says data are available “upon reasonable request”. However, the manuscript also includes PLOS One’s template text stating that authors are required to make all data underlying findings fully available. The authors need to confirm if they will be depositing a minimal anonymised data set in a repository or if they are claiming an ethical exemption.**

REPLY: Thank you. We have changed this to:

*“The dataset comprises in-depth interview transcripts from a single NHS organisation with a small and identifiable workforce, meaning that even anonymised data carry a risk of deductive disclosure. Therefore, full data sharing is restricted. Requests for access to anonymised excerpts may be considered on reasonable request, subject to appropriate governance approvals. Data access requests should be directed to the University of Bristol Research Governance Team (research-governance@bristol.ac.uk), which is the study sponsor. Requests will be considered in line with applicable ethical approvals, participant consent, and institutional data governance procedures.”* Line 595-603

**Journal Requirements:**

**When submitting your revision, we need you to address these additional requirements.**

**1. Please ensure that your manuscript meets PLOS ONE's style requirements, including those for file naming. The PLOS ONE style templates can be found at**

[**https://journals.plos.org/plosone/s/file?id=wjVg/PLOSOne_formatting_sample_main_body.pdf**](https://journals.plos.org/plosone/s/file?id=wjVg/PLOSOne_formatting_sample_main_body.pdf) **and** 
[**https://journals.plos.org/plosone/s/file?id=ba62/PLOSOne_formatting_sample_title_authors_affiliations.pdf**](https://journals.plos.org/plosone/s/file?id=ba62/PLOSOne_formatting_sample_title_authors_affiliations.pdf)

REPLY: Thank you we have amended the manuscript accordingly**.**

**2. In the ethics statement in the Methods, you have specified that verbal consent was obtained. Please provide additional details regarding how this consent was documented and witnessed, and state whether this was approved by the IRB.**

REPLY: Thank you for requesting clarification. We confirm that ethical approval for this study was granted by both the Health Research Authority (HRA) and the University Research Ethics Committee, which specifically approved the use of verbal informed consent given the remote nature of the interviews. We have amended the manuscript in the participants and sampling section of the Methods to state:

*“Given the remote interview format participants were asked to provided verbal informed consent prior to participation, which was approved by the National Health Service, Health Research Authority, Research Ethics Committee (25/HRA/2520 IRAS: 357165) and the University of Bristol Faculty of Health Sciences Research Ethics Committee (FREC) (REF: 25063), Participants were sent a participant information sheet in advance of the interview and had the opportunity to ask questions before consent was obtained. Verbal consent was obtained by DJ at the start of each interview and was audio-recorded as part of the interview recording, thereby providing a documented record of consent. Consent recordings were stored securely on encrypted institutional servers, separately from anonymised interview data. Participants were reimbursed with a shopping voucher as a thank you for their time.”* Line 111-121

**3. Thank you for stating the following in the Acknowledgments Section of your manuscript:**

**“This research was funded by the Bristol and Weston Hospitals Charity (BWHC2024-01) and supported by the National Institute for Health and Care Research Applied Research Collaboration West (NIHR ARC West), the NIHR Health Protection Research Unit (HPRU) in Evaluation and Behavioural Science (EBS) at the University of Bristol in partnership with the UK Health Security Agency (UKHSA). The views expressed in this article are those of the authors and not necessarily those of the NIHR, UKHSA or the Department of Health and Social Care.”**

**We note that you have provided funding information that is not currently declared in your Funding Statement. However, funding information should not appear in the Acknowledgments section or other areas of your manuscript. We will only publish funding information present in the Funding Statement section of the online submission form.**

**Please remove any funding-related text from the manuscript and let us know how you would like to update your Funding Statement. Currently, your Funding Statement reads as follows:**

**“This research was funded by the Bristol and Weston Hospitals Charity (BWHC-2024-01) and supported by the National Institute for Health and Care Research Applied Research Collaboration West (NIHR ARC West), the NIHR Health Protection Research Unit (HPRU) in Evaluation and Behavioural Science (EBS) at the University of Bristol in partnership with the UK Health Security Agency (UKHSA). The views expressed in this article are those of the authors and not necessarily those of the NIHR, UKHSA or the Department of Health and Social Care.”**

**Please include your amended statements within your cover letter; we will change the online submission form on your behalf.**

REPLY: Thank you. We have removed the funding statement from the manuscript and have added a request in the cover letter for the following to be added as the funding statement:

*“This research was funded by the Bristol and Weston Hospitals Charity (BWHC-2024-01) and supported by the National Institute for Health and Care Research Applied Research Collaboration West (NIHR ARC West), the NIHR Health Protection Research Unit (HPRU) in Evaluation and Behavioural Science (EBS) at the University of Bristol in partnership with the UK Health Security Agency (UKHSA). The views expressed in this article are those of the authors and not necessarily those of the NIHR, UKHSA or the Department of Health and Social Care.* The funders had no role in study design, data collection and analysis, decision to publish, or preparation of the manuscript.*”*

**4. Thank you for stating the following financial disclosure:**

**“This research was funded by the Bristol and Weston Hospitals Charity (BWHC-2024-01) and supported by the National Institute for Health and Care Research Applied Research Collaboration West (NIHR ARC West), the NIHR Health Protection Research Unit (HPRU) in Evaluation and Behavioural Science (EBS) at the University of Bristol in partnership with the UK Health Security Agency (UKHSA). The views expressed in this article are those of the authors and not necessarily those of the NIHR, UKHSA or the Department of Health and Social Care.”**

**Please state what role the funders took in the study.  If the funders had no role, please state: "The funders had no role in study design, data collection and analysis, decision to publish, or preparation of the manuscript."**

**If this statement is not correct you must amend it as needed.**

**Please include this amended Role of Funder statement in your cover letter; we will change the online submission form on your behalf.**

REPLY: Many thanks, this has been added and is now in the cover letter.

**5. In the online submission form you indicate that your data is not available for proprietary reasons and have provided a contact point for accessing this data. Please note that your current contact point is a co-author on this manuscript. According to our Data Policy, the contact point must not be an author on the manuscript and must be an institutional contact, ideally not an individual. Please revise your data statement to a non-author institutional point of contact, such as a data access or ethics committee, and send this to us via return email. Please also include contact information for the third party organization, and please include the full citation of where the data can be found.**

REPLY: Thank you. Our data availability statement is now as follows:

*“The dataset comprises in-depth interview transcripts from a single NHS organisation with a small and identifiable workforce, meaning that even anonymised data carry a risk of deductive disclosure. Therefore, full data sharing is restricted. Requests for access to anonymised excerpts may be considered on reasonable request, subject to appropriate governance approvals. Data access requests should be directed to the University of Bristol Research Governance Team (research-governance@bristol.ac.uk), which is the study sponsor. Requests will be considered in line with applicable ethical approvals, participant consent, and institutional data governance procedures”* Line 595-603

**6. We note that this data set consists of interview transcripts. Can you please confirm that all participants gave consent for interview transcript to be published?**

**If they DID provide consent for these transcripts to be published, please also confirm that the transcripts do not contain any potentially identifying information (or let us know if the participants consented to having their personal details published and made publicly available). We consider the following details to be identifying information:**

**- Names, nicknames, and initials
- Age more specific than round numbers
- GPS coordinates, physical addresses, IP addresses, email addresses
- Information in small sample sizes (e.g. 40 students from X class in X year at X university)
- Specific dates (e.g. visit dates, interview dates)
- ID numbers**

**Or, if the participants DID NOT provide consent for these transcripts to be published:**

**- Provide a de-identified version of the data or excerpts of interview responses
- Provide information regarding how these transcripts can be accessed by researchers who meet the criteria for access to confidential data, including:**

**a) the grounds for restriction
b) the name of the ethics committee, Institutional Review Board, or third-party organization that is imposing sharing restrictions on the data
c) a non-author, institutional point of contact that is able to field data access queries, in the interest of maintaining long-term data accessibility.
d) Any relevant data set names, URLs, DOIs, etc. that an independent researcher would need in order to request your minimal data set.**

**For further information on sharing data that contains sensitive participant information, please see:** [**https://journals.plos.org/plosone/s/data-availability#loc-human-research-participant-data-and-other-sensitive-data**](https://journals.plos.org/plosone/s/data-availability#loc-human-research-participant-data-and-other-sensitive-data) **If there are ethical, legal, or third-party restrictions upon your dataset, you must provide all of the following details (**[**https://journals.plos.org/plosone/s/data-availability#loc-acceptable-data-access-restrictions**](https://journals.plos.org/plosone/s/data-availability#loc-acceptable-data-access-restrictions)**):**

**1. A complete description of the dataset**

**2. The nature of the restrictions upon the data (ethical, legal, or owned by a third party) and the reasoning behind them**

**3. The full name of the body imposing the restrictions upon your dataset (ethics committee, institution, data access committee, etc)**

**4. If the data are owned by a third party, confirmation of whether the authors received any special privileges in accessing the data that other researchers would not have**

**5. Direct, non-author contact information (preferably email) for the body imposing the restrictions upon the data, to which data access requests can be sent**

**7. If the reviewer comments include a recommendation to cite specific previously published works, please review and evaluate these publications to determine whether they are relevant and should be cited. There is no requirement to cite these works unless the editor has indicated otherwise.** 

REPLY: We thank the editor for this clarification regarding data availability and participant consent. Participants provided explicit consent for their anonymised interview transcripts to be shared as restricted-access research data. Participants consented to the following statement:

*“I give permission for my anonymised interview transcript to be published on the University of Bristol Research Data Repository, data.bris, as Restricted data. I understand that this means the anonymised research data will be available to authenticated researchers, subject to a legally binding non-disclosure contract. This means it may be used for purposes not related to this study, but it will not be possible to identify me from these data”*

All transcripts from semi-structured interviews were anonymised, with direct identifiers and organisationally identifying details removed. Additional steps, including grouping participants into broad occupational categories, were taken to reduce the risk of deductive disclosure within a small and potentially identifiable NHS workforce. Due to the sensitive nature of the data and ongoing risk of deductive identification, the transcripts are not publicly available as unrestricted open-access data. Access restrictions are imposed in accordance with participant consent, ethical approvals, and University of Bristol research governance procedures. Requests for access may be considered for authenticated researchers subject to appropriate governance review and data sharing agreements.

The following data availability statement is included:

*“The dataset comprises in-depth interview transcripts from a single NHS organisation with a small and identifiable workforce, meaning that even anonymised data carry a risk of deductive disclosure. Therefore, full data sharing is restricted. Requests for access to anonymised excerpts may be considered on reasonable request, subject to appropriate governance approvals. Data access requests should be directed to the University of Bristol Research Governance Team (research-governance@bristol.ac.uk), which is the study sponsor. Requests will be considered in line with applicable ethical approvals, participant consent, and institutional data governance procedure”* Line 595-603
