# Supplementary material for: Examining the mental health support needs of NHS staff exposed to patient pregnancy and neonatal loss: A qualitative study
Source: PLoS One. 2026 Sep 17;21(9):e0358321. doi: 10.1371/journal.pone.0358321 (PMC13585218; doi:10.1371/journal.pone.0358321)
Supplement: Supplementary file 4 [file pone.0358321.s005.docx]

We thank the reviewers for their additional feedback. We have revised the manuscript in response to all comments. All changes are marked in the tracked version.

Reviewer #2: 1. Abstract section

• The abstract currently contains both an “Objective” and a “Study aims” section. These are redundant, as they essentially convey the same meaning. The authors should merge these into a single statement and instead begin the abstract with a concise background that contextualizes the study.

• The design, setting, and population details should be presented within the Methods section rather than elsewhere. Additionally, the specific qualitative approach employed must be stated.

**REPLY: Thank you, we have added a background section to the abstract and merged the information presented in the design, setting and population subsections into the methods. We have also stated the qualitative approach employed.**

**The methods section of the abstract now state:**

*This qualitative study used a reflective thematic approach. Semi-structured interviewed were conducted with 20 purposefully sampled clinical, allied and non-clinical staff from a single UK NHS trust. Interviews were audio-recorded, transcribed verbatim, anonymised and analysed using thematic analysis, with themes mapped to the study aims.*

2. Methods section
The specific qualitative approach employed must be explicitly stated to strengthen methodological transparency.

3. In Line 88 the statement “The research applied a qualitative research design” is inaccurate. Qualitative methods represent an approach rather than a design. The authors should revise this phrasing to reflect the correct methodological terminology.

**REPLY: Thank you. We have changed this to:** *This qualitative study used a reflective thematic analysis approach to explore staff experiences of pregnancy and neonatal loss. Semi-structured individual interviews were carried out with staff working in a large NHS Trust in the UK.*

4. Line 101, the comma following "short" appears misplaced and should be removed for clarity.

**REPLY: Thank you. We have removed this comma.**
