# Supplementary material for: Examining the mental health support needs of NHS staff exposed to patient pregnancy and neonatal loss: A qualitative study
Source: PLoS One. 2026 Sep 17;21(9):e0358321. doi: 10.1371/journal.pone.0358321 (PMC13585218; doi:10.1371/journal.pone.0358321)
Supplement: Supplementary file 5 [file pone.0358321.s006.docx]

We thank the reviewers for their additional feedback. Looking at the clean version submitted for the second round of revisions, the below requested revisions have already been addressed. We are therefore resubmitting the clean version that was submitted for revision #2 with highlights to mark the changes requested below.

1. **Methods section: The specific qualitative approach employed needs to be explicitly stated for methodological transparency, both in the abstract and the main Methods section.**

**REPLY: This is stated in the abstract on line 24 and in the methods section in the design and setting sub section (line 83)**

2. **Terminology correction (Line 88): The statement "The research applied a qualitative research design" is imprecise, as qualitative research is more accurately described as an approach rather than a design.**

**REPLY: As above. This is stated on line 24 and 83**
