# Supplementary material for: Examining the mental health support needs of NHS staff exposed to patient pregnancy and neonatal loss: A qualitative study
Source: PLoS One. 2026 Sep 17;21(9):e0358321. doi: 10.1371/journal.pone.0358321 (PMC13585218; doi:10.1371/journal.pone.0358321)
Supplement: Peer review history

# Peer review: Decision Letter 0

**DOI:** 10.1371/journal.pone.0358321.r001

**Authors:** Dorothy Boakye

25 May 2026

Dear Dr. Harrison,

Thank you for submitting your manuscript to PLOS ONE. After careful consideration, we feel that it has merit but does not fully meet PLOS ONE’s publication criteria as it currently stands. Therefore, we invite you to submit a revised version of the manuscript that addresses the points raised during the review process.

Please submit your revised manuscript by Jul 09 2026 11:59PM. If you will need more time than this to complete your revisions, please reply to this message or contact the journal office at plosone@plos.org. When you're ready to submit your revision, log on to https://www.editorialmanager.com/pone/ and select the 'Submissions Needing Revision' folder to locate your manuscript file.

- A letter that responds to each point raised by the academic editor and reviewer(s). You should upload this letter as a separate file labeled 'Response to Reviewers'.
- A marked-up copy of your manuscript that highlights changes made to the original version. You should upload this as a separate file labeled 'Revised Manuscript with Track Changes'.
- An unmarked version of your revised paper without tracked changes. You should upload this as a separate file labeled 'Manuscript'.

If you would like to make changes to your financial disclosure, please include your updated statement in your cover letter. Guidelines for resubmitting your figure files are available below the reviewer comments at the end of this letter.

If applicable, we recommend that you deposit your laboratory protocols in protocols.io to enhance the reproducibility of your results. Protocols.io assigns your protocol its own identifier (DOI) so that it can be cited independently in the future. For instructions see: https://journals.plos.org/plosone/s/submission-guidelines#loc-laboratory-protocols. Additionally, PLOS ONE offers an option for publishing peer-reviewed Lab Protocol articles, which describe protocols hosted on protocols.io. Read more information on sharing protocols at https://plos.org/protocols?utm_medium=editorial-email&utm_source=authorletters&utm_campaign=protocols.

As the corresponding author, your ORCID iD is verified in the submission system and will appear in the published article. PLOS supports the use of ORCID, and we encourage all coauthors to register for an ORCID iD and use it as well. Please encourage your coauthors to verify their ORCID iD within the submission system before final acceptance, as unverified ORCID iDs will not appear in the published article. *Only*  the individual author can complete the verification step; PLOS staff *cannot*  verify ORCID iDs on behalf of authors.

We look forward to receiving your revised manuscript.

Kind regards,

Dorothy Serwaa Boakye, PGDE,MA,Mphil, RN, BSc

Academic Editor

PLOS One

Journal Requirements:

When submitting your revision, we need you to address these additional requirements.

1. Please ensure that your manuscript meets PLOS ONE's style requirements, including those for file naming. The PLOS ONE style templates can be found at

https://journals.plos.org/plosone/s/file?id=wjVg/PLOSOne_formatting_sample_main_body.pdf and

https://journals.plos.org/plosone/s/file?id=ba62/PLOSOne_formatting_sample_title_authors_affiliations.pdf

2. In the ethics statement in the Methods, you have specified that verbal consent was obtained. Please provide additional details regarding how this consent was documented and witnessed, and state whether this was approved by the IRB.

3. Thank you for stating the following in the Acknowledgments Section of your manuscript:

“This research was funded by the Bristol and Weston Hospitals Charity (BWHC2024-01) and supported by the National Institute for Health and Care Research Applied Research Collaboration West (NIHR ARC West), the NIHR Health Protection Research Unit (HPRU) in Evaluation and Behavioural Science (EBS) at the University of Bristol in partnership with the UK Health Security Agency (UKHSA). The views expressed in this article are those of the authors and not necessarily those of the NIHR, UKHSA or the Department of Health and Social Care.”

We note that you have provided funding information that is not currently declared in your Funding Statement. However, funding information should not appear in the Acknowledgments section or other areas of your manuscript. We will only publish funding information present in the Funding Statement section of the online submission form.

Please remove any funding-related text from the manuscript and let us know how you would like to update your Funding Statement. Currently, your Funding Statement reads as follows:

“This research was funded by the Bristol and Weston Hospitals Charity (BWHC-2024-01) and supported by the National Institute for Health and Care Research Applied Research Collaboration West (NIHR ARC West), the NIHR Health Protection Research Unit (HPRU) in Evaluation and Behavioural Science (EBS) at the University of Bristol in partnership with the UK Health Security Agency (UKHSA). The views expressed in this article are those of the authors and not necessarily those of the NIHR, UKHSA or the Department of Health and Social Care.”

Please include your amended statements within your cover letter; we will change the online submission form on your behalf.

4. Thank you for stating the following financial disclosure:

“This research was funded by the Bristol and Weston Hospitals Charity (BWHC-2024-01) and supported by the National Institute for Health and Care Research Applied Research Collaboration West (NIHR ARC West), the NIHR Health Protection Research Unit (HPRU) in Evaluation and Behavioural Science (EBS) at the University of Bristol in partnership with the UK Health Security Agency (UKHSA). The views expressed in this article are those of the authors and not necessarily those of the NIHR, UKHSA or the Department of Health and Social Care.”

Please state what role the funders took in the study.  If the funders had no role, please state: "The funders had no role in study design, data collection and analysis, decision to publish, or preparation of the manuscript."

If this statement is not correct you must amend it as needed.

Please include this amended Role of Funder statement in your cover letter; we will change the online submission form on your behalf.

5. In the online submission form you indicate that your data is not available for proprietary reasons and have provided a contact point for accessing this data. Please note that your current contact point is a co-author on this manuscript. According to our Data Policy, the contact point must not be an author on the manuscript and must be an institutional contact, ideally not an individual. Please revise your data statement to a non-author institutional point of contact, such as a data access or ethics committee, and send this to us via return email. Please also include contact information for the third party organization, and please include the full citation of where the data can be found.

6. We note that this data set consists of interview transcripts. Can you please confirm that all participants gave consent for interview transcript to be published?

If they DID provide consent for these transcripts to be published, please also confirm that the transcripts do not contain any potentially identifying information (or let us know if the participants consented to having their personal details published and made publicly available). We consider the following details to be identifying information:

- Names, nicknames, and initials

- Age more specific than round numbers

- GPS coordinates, physical addresses, IP addresses, email addresses

- Information in small sample sizes (e.g. 40 students from X class in X year at X university)

- Specific dates (e.g. visit dates, interview dates)

- ID numbers

Or, if the participants DID NOT provide consent for these transcripts to be published:

- Provide a de-identified version of the data or excerpts of interview responses

- Provide information regarding how these transcripts can be accessed by researchers who meet the criteria for access to confidential data, including:

a) the grounds for restriction

b) the name of the ethics committee, Institutional Review Board, or third-party organization that is imposing sharing restrictions on the data

c) a non-author, institutional point of contact that is able to field data access queries, in the interest of maintaining long-term data accessibility.

d) Any relevant data set names, URLs, DOIs, etc. that an independent researcher would need in order to request your minimal data set.

For further information on sharing data that contains sensitive participant information, please see: https://journals.plos.org/plosone/s/data-availability#loc-human-research-participant-data-and-other-sensitive-data

If there are ethical, legal, or third-party restrictions upon your dataset, you must provide all of the following details (https://journals.plos.org/plosone/s/data-availability#loc-acceptable-data-access-restrictions):

1. A complete description of the dataset

2. The nature of the restrictions upon the data (ethical, legal, or owned by a third party) and the reasoning behind them

3. The full name of the body imposing the restrictions upon your dataset (ethics committee, institution, data access committee, etc)

4. If the data are owned by a third party, confirmation of whether the authors received any special privileges in accessing the data that other researchers would not have

5. Direct, non-author contact information (preferably email) for the body imposing the restrictions upon the data, to which data access requests can be sent

7. If the reviewer comments include a recommendation to cite specific previously published works, please review and evaluate these publications to determine whether they are relevant and should be cited. There is no requirement to cite these works unless the editor has indicated otherwise.

[Note: HTML markup is below. Please do not edit.]

Reviewers' comments:

Reviewer's Responses to Questions

**Comments to the Author**

1. Is the manuscript technically sound, and do the data support the conclusions?

Reviewer #1: Yes

Reviewer #2: Yes

**********

2. Has the statistical analysis been performed appropriately and rigorously?

Reviewer #1: Yes

Reviewer #2: Yes

**********

3. Have the authors made all data underlying the findings in their manuscript fully available?

The PLOS Data policy

Reviewer #1: Yes

Reviewer #2: No

**********

4. Is the manuscript presented in an intelligible fashion and written in standard English?

Reviewer #1: Yes

Reviewer #2: Yes

**********

Reviewer #1: MAJOR COMMENTS

1. Methodological Framework and Analytical Rigour

The authors state thematic analysis (Braun & Clarke) was used, but describe an approach that is inconsistent with a fully reflexive stance. Themes are explicitly "mapped to the study aims," which reflects a more framework-driven or deductive approach. This tension is not acknowledged or justified. The authors should either: (a) transparently describe their approach as a hybrid or directed thematic analysis, or (b) explain how inductive theme generation was reconciled with aim-mapping without compromising analytical integrity.

2. Insufficient Reporting of Reflexivity

Reflexivity is poorly addressed. The study involves a single interviewer (DJ) who is also a researcher embedded in the institutional setting. No positionality statement is provided, and there is no discussion of how the team's professional backgrounds, institutional affiliations, or personal experiences may have shaped data collection and analysis. This is a fundamental requirement for qualitative research and must be added.

3. Saturation and Sample Justification

The authors recruited 20 of 49 volunteers via purposive sampling but provide no rationale for why 20 participants were sufficient. There is no discussion of data saturation or information power. Given the heterogeneous sample (seven departments, three staff categories, varied seniority), the adequacy of the sample for capturing the full range of experiences warrants explicit justification.

4. Consent Reporting Inconsistency

The methods section states participants provided "verbal informed consent," while the ethics statement to the journal says "informed consent" without specifying the form. For a study involving sensitive topics and audio recording, verbal-only consent requires explicit justification. If written consent was also obtained, this should be clarified. PLOS ONE requires precision on this point.

5. Reference Error — Reference 8

Reference 8, cited to support emotional distress and burnout in O&G staff, is incorrectly cited. The reference listed is Lisy et al. (2018) on "lesbian, gay, and bisexual people with cancer care" — entirely unrelated to the claim it supports. This must be corrected with an appropriate citation.

6. Data Availability Statement

PLOS ONE requires that data underlying findings be made "fully available and without restriction" unless a specific exemption applies. The statement that data are "available from the corresponding author upon reasonable request" does not meet this requirement. The authors must deposit anonymised data in an appropriate repository or provide a detailed justification for restriction (e.g., participant confidentiality, ethics committee prohibition).

MINOR COMMENTS

7. Abstract Formatting

Theme (1) in the Results ("nobody prepared me for this") is not capitalised, inconsistent with the other themes. Capitalisation should be standardised throughout the abstract and manuscript.

8. Typographical and Grammatical Errors

Several minor errors require correction: (a) "its'" (p. 15) should be "its"; (b) a comma splice appears in the Conclusion ("support., including"); (c) "Bristol Bristol" appears twice in the affiliations list and should be corrected; (d) missing comma after aim (3) in the Introduction ("3) support previously used 4) support needed or wanted").

9. Theme Overlap Between Themes 3 and 4

Themes 3 (peer support vs. organisational support) and 4 (reliance on informal support networks) are conceptually overlapping. The distinction between "perception of support available" and "support previously used" as organising frames is understandable, but the narrative presentation blurs this boundary. The authors should sharpen the conceptual distinction and ensure each theme presents genuinely distinct analytical content.

10. Table 2 — Recommendations

The 13 recommendations in Table 2 are useful practically but are largely descriptive and not sufficiently grounded in the data. Each recommendation should be explicitly linked to a specific finding or theme. Additionally, the label "Gold standard" is not justified by the evidence base from a single-site qualitative study and should be revised to something more appropriately qualified (e.g., "Evidence-informed recommendations").

11. Transferability

The authors acknowledge single-site limitations but do not sufficiently discuss transferability. A brief reflection on which findings are likely context-specific to tertiary, high-complexity care settings versus those that may generalise to other NHS trusts would strengthen the discussion.

12. Missing Reporting Checklist

The manuscript does not appear to reference or append a qualitative reporting checklist (e.g., COREQ or SRQR). PLOS ONE strongly encourages the use of reporting guidelines for qualitative studies. Authors should complete and submit the relevant checklist.

ADDITIONAL COMMENTS ON PRESENTATION AND RESULTS REPORTING

13. Absence of Subthemes

Six broad themes are presented, but none are developed with subthemes despite the richness and heterogeneity of the data evident in the quotes. For example, Theme 2 ("Reaching a tipping point") encompasses at least three distinct experiential dimensions — cumulative shift-based fatigue, personal fertility/loss history compounding professional distress, and seniority-related differences in resilience — yet these are collapsed into a single undifferentiated theme narrative. Similarly, Theme 6 ("One size does not fit all") bundles training needs, structural support preferences, timing of support, and privacy concerns without analytical distinction. The absence of subthemes limits the depth and precision of the analysis and understates the complexity of participants' experiences. The authors should consider developing an explicit thematic hierarchy with clearly labelled subthemes under each main theme, accompanied by brief descriptions of how subthemes relate to the overarching theme.

14. Illustrative Quotes Sequestered in a Table Rather Than Integrated into the Narrative

The decision to present all illustrative quotations in a standalone table (Table 1) rather than embedding them within the thematic narrative is a significant presentational weakness with analytical consequences. In qualitative research, quotes serve not merely as illustrations but as the evidential foundation for interpretive claims. Separating them from the narrative text prevents readers from evaluating the interpretive logic — that is, from judging whether the authors' analytical claims are warranted by the data. As presented, readers must cross-reference between the narrative and the table, disrupting the reading flow and obscuring the relationship between data and interpretation. This is not standard practice in qualitative thematic analysis reporting. The authors should embed relevant quotes directly within the thematic narrative at the point where the interpretive claim is made, following the established convention of: claim → quote → interpretive commentary. A supplementary table may be retained if desired, but it cannot substitute for in-text integration of evidence.

Reviewer #2: This review examines the manuscript titled “Examining the mental health support needs of NHS staff exposed to patient pregnancy and neonatal loss: a qualitative study” (PONE-D-26-16902).

The paper addresses a gap in the literature by including allied and non-clinical staff, who are often overlooked in research concerning perinatal loss.

The study is well-grounded and methodologically sound. However, the following areas require further attention:

1. Abstract

Line 32: The abstract lists “Experiences of exposure,” “perceived support,” etc., as “Main outcome measures”. In qualitative research, these are typically referred to as research questions or study aims rather than “outcome measures,” which is a term more suited to quantitative or clinical trials.

Line 44: The conclusion in the abstract is somewhat generic. It would benefit from highlighting the specific “tipping point” concept or the unique vulnerability of allied/support staff identified in the results.

2. Methodology

Sampling

• The authors recruited 20 participants across a wide array of roles: doctors, nurses, nursing assistants, theatre staff, sonographers, administrators, and midwives. While purposive sampling was used, it is unclear if thematic saturation was reached for each of these distinct groups. For example, only one administrative staff member was interviewed. The authors should clarify whether the findings for these smaller subgroups are sufficiently robust or should be presented as preliminary.

• The manuscript mentions an “iterative approach” to the topic guide and sampling. Providing a brief example of how the topic guide evolved based on early interviews would strengthen the “Methods” section and demonstrate analytical rigour.

Consent Procedures

• The text notes that participants provided verbal informed consent (line 100). The authors should explicitly state if this was approved by the Ethics Committee instead of written consent, and how this verbal consent was recorded or witnessed to meet standard ethical reporting guidelines.

3. Results

• There is significant conceptual overlap between Theme 3 (Peer support versus inconsistent organisational support) and Theme 4 (Reliance on informal support networks). Theme 4 essentially explores the usage of what Theme 3 identifies as available. The authors should consider merging these or more clearly distinguishing the “perception” of support from the “behavioural reliance” on it.

• To protect anonymity, roles were grouped into ‘clinical’, ‘allied’, and ‘support’. However, this occasionally obscures important context. For instance, the “feticide” mentioned as a stressor is likely specific to a few medical roles. The authors should ensure that by grouping, they don’t lose the nuance of how specific clinical tasks impact mental health.

4. Discussion

• The concept of a “tipping point” is a major finding. The discussion would be strengthened by linking this more clearly to existing theories of secondary traumatic stress or compassion fatigue, which are mentioned in the introduction but are less integrated into the interpretation of the “tipping point”.

• The study was conducted in a single NHS Trust that provides tertiary care. The authors correctly identify this as a limitation, but they should further discuss how the “high-risk, complex” nature of this specific trust might have skewed the results compared to a smaller community-based maternity unit.

5. Recommendations (Table 2)

• Table 2 provides 13 “gold standard” recommendations. While comprehensive, this list is quite long for a healthcare lead to implement. The authors should consider categorising these (e.g., Immediate vs. Long-term, or Local vs. Organisational) or identifying the “top three” most critical interventions based on participant feedback.

• The study advocates for co-designed support tools. It would be helpful to briefly discuss the practical barriers to co-design in a high-pressure NHS environment (e.g., staff time and funding) and how they might be mitigated.

6. Minor/Technical Issues

• The current statement says data are available “upon reasonable request”. However, the manuscript also includes PLOS One’s template text stating that authors are required to make all data underlying findings fully available. The authors need to confirm if they will be depositing a minimal anonymised data set in a repository or if they are claiming an ethical exemption.

**********

what does this mean?). If published, this will include your full peer review and any attached files.

If you choose “no”, your identity will remain anonymous but your review may still be made public.

**Do you want your identity to be public for this peer review?**  For information about this choice, including consent withdrawal, please see our Privacy Policy

Reviewer #1: No

Reviewer #2: No

**********

[NOTE: If reviewer comments were submitted as an attachment file, they will be attached to this email and accessible via the submission site. Please log into your account, locate the manuscript record, and check for the action link "View Attachments". If this link does not appear, there are no attachment files.]

To ensure your figures meet our technical requirements, please review our figure guidelines: https://journals.plos.org/plosone/s/figures

You may also use PLOS’s free figure tool, NAAS, to help you prepare publication quality figures: https://journals.plos.org/plosone/s/figures#loc-tools-for-figure-preparation.

NAAS will assess whether your figures meet our technical requirements by comparing each figure against our figure specifications.

# Author comment: Author response to Decision Letter 1

**DOI:** 10.1371/journal.pone.0358321.r002

11 Jun 2026

We thank the editors and reviewers for their constructive and detailed feedback. We have revised the manuscript in response to all comments. All changes are marked in the tracked version. Line numbers refer to the manuscript with tracked changes.

Reviewer #1: MAJOR COMMENTS

1. Methodological Framework and Analytical Rigour

The authors state thematic analysis (Braun & Clarke) was used, but describe an approach that is inconsistent with a fully reflexive stance. Themes are explicitly "mapped to the study aims," which reflects a more framework-driven or deductive approach. This tension is not acknowledged or justified. The authors should either: (a) transparently describe their approach as a hybrid or directed thematic analysis, or (b) explain how inductive theme generation was reconciled with aim-mapping without compromising analytical integrity.

REPLY: Thank you to the reviewer for this important observation. We agree that our original wording did not sufficiently distinguish between the inductive development of themes and the later stage of relating findings back to the study aims. We have revised the Methods section to clarify that the analysis followed an iterative and reflexive thematic analysis approach, with themes generated inductively from the data. We have highted that our approach deviates from Braun and Clarke’s as the themes have been interpreted in relation to the research aims during the write-up phase and presented in such a way to aid future intervention development. We have also clarified the collaborative and reflexive nature of the analytic process. The methods section now states:

“Interviews were audio recorded and transcribed verbatim by a professional transcription service. NVivo software (QSR International Pty Ltd.,) was used to manage and analyse data using thematic analysis (20) following an iterative, interpretive, and reflexive approach. Transcripts were read several times for familiarisation, and initial codes were generated inductively by DJ to capture meaningful features of the data. CH independently read and coded a substantial proportion of the transcripts, contributing alternative interpretations, supporting reflexivity and the development of a richer, more nuanced analysis. Throughout the analysis, DJ and CH regularly met with SN (Consultant Obstetrician and Subspecialist in Fetal/Maternal Medicine), MH (Lead Neonatal Nurse for Bereavement), HF (Psychologist and Behavioural Scientist with expertise in qualitative research) to reflect on assumptions and discuss analytic decisions while considering how differing perspectives and backgrounds may have shaped interpretation of the data. Sub-themes were developed iteratively through close engagement with the dataset and were generated from patterns identified across participant accounts rather than being predetermined by the study aims. Subthemes were further refined and organised into broader overarching themes. Deviating from Braun & Clarke’s approach to reporting findings, the themes were organised in relation to the aims of the research to support coherent presentation and interpretation of the findings which are intended to inform future intervention development” Line 129-148.

2. Insufficient Reporting of Reflexivity

Reflexivity is poorly addressed. The study involves a single interviewer (DJ) who is also a researcher embedded in the institutional setting. No positionality statement is provided, and there is no discussion of how the team's professional backgrounds, institutional affiliations, or personal experiences may have shaped data collection and analysis. This is a fundamental requirement for qualitative research and must be added.

REPLY: Thank you for highlighting the need for greater reflexive transparency. We agree that the original manuscript did not provide sufficient detail regarding the researcher’s positionality. We have now added a reflexive statement in the methos section describing DJs role in addition to highlighting consideration given to the potential influence of her positioning on participant responses and interview dynamics. In the methods section it now states:

“Interviews were carried out online (MS Teams) by DJ (a research associate with a background in health research including topics related to pregnancy, breastfeeding and perinatal mental health in addition to previous midwifery experience). A flexible topic guide, informed by the aims of the study, was developed and adapted appropriately for the participant’s job role. Reflexive consideration was given to how DJs positioning may have influenced participant responses and interview dynamics”. Lines 122-128

We have also added positioning for the other researchers involved in the analysis:

“CH (a behavioural scientist with expertise in sexual and reproductive health) independently read and coded a substantial proportion of the transcripts, contributing alternative interpretations, supporting reflexivity and the development of a richer, more nuanced analysis. Throughout the analysis, DJ and CH regularly met with SN (Consultant Obstetrician and Subspecialist in Fetal/Maternal Medicine), MH (Lead Neonatal Nurse for Bereavement), HF (Psychologist and Behavioural Scientist with expertise in qualitative research) to reflect on assumptions and discuss analytic decisions while considering how differing perspectives and backgrounds may have shaped interpretation of the data. Sub-themes were developed iteratively through close engagement with the dataset and were generated from patterns identified across participant accounts rather than being predetermined by the study aims. Subthemes were further refined and organised into broader overarching themes. Deviating from Braun & Clarke’s approach to reporting findings the themes were organised in relation to the aims of the research to support coherent presentation and interpretation of the findings which are intended to inform future intervention development”. Lines 134-148

3. Saturation and Sample Justification

The authors recruited 20 of 49 volunteers via purposive sampling but provide no rationale for why 20 participants were sufficient. There is no discussion of data saturation or information power. Given the heterogeneous sample (seven departments, three staff categories, varied seniority), the adequacy of the sample for capturing the full range of experiences warrants explicit justification.

REPLY: Thank you. We thank the reviewer for this important observation. The study aimed to capture a breadth of perspectives across staff groups exposed to pregnancy and neonatal loss rather than to achieve thematic saturation within each individual professional subgroup. Whilst we acknowledge we deviated from Braun & Clarke’s approach to reporting themes; we maintained commitment to their approach through the rejection of the concept of saturation which they position as a positivist approach to qualitative research (please see: Braun & Clarke (2021) https://doi.org/10.1080/2159676X.2019.1704846\). Recruitment was voluntary, and although purposive sampling was used to achieve representation across clinical, allied, support, and administrative roles, participation was dependent on staff expressing interest in taking part. As a result, some groups were represented by smaller numbers of participants, including administrative staff, where only one staff member volunteered to participate.

The sample size was also shaped by the resources available to support recruitment, data collection, transcription, and analysis. Given the exploratory qualitative design, the intention was not to generate profession-specific findings, but rather to identify shared and contrasting experiences across a range of staff groups exposed to pregnancy and neonatal loss within the organisation.

We have added the following to the strengths and limitations section, to acknowledge this potential limitation:

“Although purposive sampling was used to achieve representation across a range of clinical, allied, support, and administrative roles, participation was voluntary and recruitment was therefore dependent on staff willingness to take part. As a result, some staff groups were represented by smaller numbers of participants than others; for example, only one administrative staff member participated despite efforts to recruit across all relevant roles. The study was designed to explore shared and contrasting experiences across the workforce therefore findings relating to smaller staff groups should be interpreted with appropriate caution. However, despite smaller numbers in some roles, there was substantial consistency in the patterns of experience described across participant accounts, and no substantially new themes emerged in later interviews, suggesting sufficient depth and repetition of data to support the analytic findings” Lines 551-561

4. Consent Reporting Inconsistency

The methods section states participants provided "verbal informed consent," while the ethics statement to the journal says "informed consent" without specifying the form. For a study involving sensitive topics and audio recording, verbal-only consent requires explicit justification. If written consent was also obtained, this should be clarified. PLOS ONE requires precision on this point.

REPLY: Apologies for this oversight, we have now specified in the ethics statement for the journal that verbal informed consent was obtained. We now have the following included in the participants and sampling section of the manuscript as per journal requirements highlighted above:

“Given the remote interview format participants were asked to provided verbal informed consent prior to participation, which was approved by the National Health Service, Health Research Authority, Research Ethics Committee (25/HRA/2520 IRAS: 357165) and the University of Bristol Faculty of Health Sciences Research Ethics Committee (FREC) (REF: 25063), Participants were sent a participant information sheet in advance of the interview and had the opportunity to ask questions before consent was obtained. Verbal consent was obtained by DJ at the start of each interview and was audio-recorded as part of the interview recording, thereby providing a documented record of consent. Consent recordings were stored securely on encrypted institutional servers, separately from anonymised interview data. Participants were reimbursed with a shopping voucher as a thank you for their time.” Line 111-121

5. Reference Error — Reference 8

Reference 8, cited to support emotional distress and burnout in O&G staff, is incorrectly cited. The reference listed is Lisy et al. (2018) on "lesbian, gay, and bisexual people with cancer care" — entirely unrelated to the claim it supports. This must be corrected with an appropriate citation.

REPLY: Apologies for this error. We have now replaced the reference.

6. Data Availability Statement

PLOS ONE requires that data underlying findings be made "fully available and without restriction" unless a specific exemption applies. The statement that data are "available from the corresponding author upon reasonable request" does not meet this requirement. The authors must deposit anonymised data in an appropriate repository or provide a detailed justification for restriction (e.g., participant confidentiality, ethics committee prohibition).

REPLY: We thank the reviewer for highlighting the journal’s data availability requirements. We recognise the importance of transparency and open data principles. However, in this study full anonymisation of the qualitative dataset is not sufficient to guarantee participant confidentiality. In line with PLOS ONE data sharing policies, we have revised the Data Availability Statement to reflect this:

“The dataset comprises in-depth interview transcripts from a single NHS organisation with a small and identifiable workforce, meaning that even anonymised data carry a risk of deductive disclosure. Therefore, full data sharing is restricted. Requests for access to anonymised excerpts may be considered on reasonable request, subject to appropriate governance approvals. Data access requests should be directed to the University of Bristol Research Governance Team (research-governance@bristol.ac.uk), which is the study sponsor. Requests will be considered in line with applicable ethical approvals, participant consent, and institutional data governance procedures.” Line 595-603

MINOR COMMENTS

7. Abstract Formatting

Theme (1) in the Results ("nobody prepared me for this") is not capitalised, inconsistent with the other themes. Capitalisation should be standardised throughout the abstract and manuscript.

REPLY: Many thanks. We have amended so all themes are not capitalised.

8. Typographical and Grammatical Errors

Several minor errors require correction: (a) "its'" (p. 15) should be "its"; (b) a comma splice appears in the Conclusion ("support., including"); (c) "Bristol Bristol" appears twice in the affiliations list and should be corrected; (d) missing comma after aim (3) in the Introduction ("3) support previously used 4) support needed or wanted").

REPLY: Thank you for highlighting these errors. They have now been addressed.

9. Theme Overlap Between Themes 3 and 4

Themes 3 (peer support vs. organisational support) and 4 (reliance on informal support networks) are conceptually overlapping. The distinction between "perception of support available" and "support previously used" as organising frames is understandable, but the narrative presentation blurs this boundary. The authors should sharpen the conceptual distinction and ensure each theme presents genuinely distinct analytical content.

REPLY: REPLY: Thank you. We have revised the manuscript in two ways to improve conceptual clarity. Firstly, we have retained both themes but clarified their distinct focus by adding the following statement at the beginning of theme 4: “While theme 3 describes participants perceptions and awareness of available support, this theme focuses on the support that staff actually accessed and relied upon in practice”. Second, we have refined the thematic structure by introducing subthemes as suggested by reviewer 1. Together we feel these revisions improve clarity.

10. Table 2 — Recommendations

The 13 recommendations in Table 2 are useful practically but are largely descriptive and not sufficiently grounded in the data. Each recommendation should be explicitly linked to a specific finding or theme. Additionally, the label "Gold standard" is not justified by the evidence base from a single-site qualitative study and should be revised to something more appropriately qualified (e.g., "Evidence-informed recommendations").

REPLY: Thank you. We have changed this to “Evidence-informed recommendations” as recommended.

We have also added a column to show the theme/s from which the recommendations derive and structured Table 2 in line with suggestions from reviewer 2.

11. Transferability

The authors acknowledge single-site limitations but do not sufficiently discuss transferability. A brief reflection on which findings are likely context-specific to tertiary, high-complexity care settings versus those that may generalise to other NHS trusts would strengthen the discussion.

REPLY: Thank you for this helpful suggestion. We have expanded the strengths and limitations section to provide greater consideration of transferability. We have included:

“The Trust provides high-complexity maternity, fetal medicine neonatal intensive care and bereavement services. As a result, staff are more likely to encounter complex, high-acuity cases and repeated exposure to pregnancy and neonatal loss compared with staff working in smaller district general hospitals. This may have contributed to the intensity and frequency of emotional exposure described by participants, and therefore the reported experience of cumulative burden and tipping points may be more pronounced than in less specialised settings. However, several findings are likely to have relevance across other NHS settings where staff are exposed to pregnancy and neonatal loss. In particular, the cumulative emotional burden associated with repeated exposure to distressing events, reliance on informal peer support, variability in preparedness and inconsistent awarenes

Attachment Submitted filename: Response to reviewer comments_R1_10_06_26.docx

# Peer review: Decision Letter 1

**DOI:** 10.1371/journal.pone.0358321.r003

**Authors:** Dorothy Boakye

22 Jul 2026

Dear Dr. Harrison,

Please submit your revised manuscript by Sep 05 2026 11:59PM. If you will need more time than this to complete your revisions, please reply to this message or contact the journal office at plosone@plos.org. When you're ready to submit your revision, log on to https://www.editorialmanager.com/pone/ and select the 'Submissions Needing Revision' folder to locate your manuscript file.

- A letter that responds to each point raised by the academic editor and reviewer(s). You should upload this letter as a separate file labeled 'Response to Reviewers'.
- A marked-up copy of your manuscript that highlights changes made to the original version. You should upload this as a separate file labeled 'Revised Manuscript with Track Changes'.
- An unmarked version of your revised paper without tracked changes. You should upload this as a separate file labeled 'Manuscript'.

If you would like to make changes to your financial disclosure, please include your updated statement in your cover letter. Guidelines for resubmitting your figure files are available below the reviewer comments at the end of this letter.

If applicable, we recommend that you deposit your laboratory protocols in protocols.io to enhance the reproducibility of your results. Protocols.io assigns your protocol its own identifier (DOI) so that it can be cited independently in the future. For instructions see: https://journals.plos.org/plosone/s/submission-guidelines#loc-laboratory-protocols. Additionally, PLOS ONE offers an option for publishing peer-reviewed Lab Protocol articles, which describe protocols hosted on protocols.io. Read more information on sharing protocols at https://plos.org/protocols?utm_medium=editorial-email&utm_source=authorletters&utm_campaign=protocols.

As the corresponding author, your ORCID iD is verified in the submission system and will appear in the published article. PLOS supports the use of ORCID, and we encourage all coauthors to register for an ORCID iD and use it as well. Please encourage your coauthors to verify their ORCID iD within the submission system before final acceptance, as unverified ORCID iDs will not appear in the published article. *Only*  the individual author can complete the verification step; PLOS staff *cannot*  verify ORCID iDs on behalf of authors.

We look forward to receiving your revised manuscript.

Kind regards,

Dorothy Serwaa Boakye, PGDE,MA,Mphil, RN, BSc

Academic Editor

PLOS One

Journal Requirements:

If the reviewer comments include a recommendation to cite specific previously published works, please review and evaluate these publications to determine whether they are relevant and should be cited. There is no requirement to cite these works unless the editor has indicated otherwise.

Please review your reference list to ensure that it is complete and correct. If you have cited papers that have been retracted, please include the rationale for doing so in the manuscript text, or remove these references and replace them with relevant current references. Any changes to the reference list should be mentioned in the rebuttal letter that accompanies your revised manuscript. If you need to cite a retracted article, indicate the article’s retracted status in the References list and also include a citation and full reference for the retraction notice.

[Note: HTML markup is below. Please do not edit.]

Reviewers' comments:

Reviewer's Responses to Questions

**Comments to the Author**

Reviewer #1: All comments have been addressed

Reviewer #2: All comments have been addressed

**********

2. Is the manuscript technically sound, and do the data support the conclusions?

Reviewer #1: Yes

Reviewer #2: Yes

**********

3. Has the statistical analysis been performed appropriately and rigorously?

Reviewer #1: N/A

Reviewer #2: Yes

**********

4. Have the authors made all data underlying the findings in their manuscript fully available?

The PLOS Data policy

Reviewer #1: Yes

Reviewer #2: Yes

**********

5. Is the manuscript presented in an intelligible fashion and written in standard English?

Reviewer #1: Yes

Reviewer #2: Yes

**********

Reviewer #1: substantively, this is a strong, comprehensive revision. Nearly every scientific and methodological point was properly incorporated into the manuscript, not just asserted in the letter.

Reviewer #2: 1. Abstract section

• The abstract currently contains both an “Objective” and a “Study aims” section. These are redundant, as they essentially convey the same meaning. The authors should merge these into a single statement and instead begin the abstract with a concise background that contextualizes the study.

• The design, setting, and population details should be presented within the Methods section rather than elsewhere. Additionally, the specific qualitative approach employed must be stated.

2. Methods section

The specific qualitative approach employed must be explicitly stated to strengthen methodological transparency.

3. In Line 88 the statement “The research applied a qualitative research design” is inaccurate. Qualitative methods represent an approach rather than a design. The authors should revise this phrasing to reflect the correct methodological terminology.

4. Line 101, the comma following "short" appears misplaced and should be removed for clarity.

**********

what does this mean?). If published, this will include your full peer review and any attached files.

If you choose “no”, your identity will remain anonymous but your review may still be made public.

**Do you want your identity to be public for this peer review?**  For information about this choice, including consent withdrawal, please see our Privacy Policy

Reviewer #1: No

Reviewer #2: No

**********

[NOTE: If reviewer comments were submitted as an attachment file, they will be attached to this email and accessible via the submission site. Please log into your account, locate the manuscript record, and check for the action link "View Attachments". If this link does not appear, there are no attachment files.]

To ensure your figures meet our technical requirements, please review our figure guidelines: https://journals.plos.org/plosone/s/figures

You may also use PLOS’s free figure tool, NAAS, to help you prepare publication quality figures: https://journals.plos.org/plosone/s/figures#loc-tools-for-figure-preparation.

NAAS will assess whether your figures meet our technical requirements by comparing each figure against our figure specifications.

# Author comment: Author response to Decision Letter 2

**DOI:** 10.1371/journal.pone.0358321.r004

22 Jul 2026

We thank the reviewers for their additional feedback. We have revised the manuscript in response to all comments. All changes are marked in the tracked version.

Reviewer #2: 1. Abstract section

• The abstract currently contains both an “Objective” and a “Study aims” section. These are redundant, as they essentially convey the same meaning. The authors should merge these into a single statement and instead begin the abstract with a concise background that contextualizes the study.

• The design, setting, and population details should be presented within the Methods section rather than elsewhere. Additionally, the specific qualitative approach employed must be stated.

REPLY: Thank you, we have added a background section to the abstract and merged the information presented in the design, setting and population subsections into the methods. We have also stated the qualitative approach employed.

The methods section of the abstract now state:

This qualitative study used a reflective thematic approach. Semi-structured interviewed were conducted with 20 purposefully sampled clinical, allied and non-clinical staff from a single UK NHS trust. Interviews were audio-recorded, transcribed verbatim, anonymised and analysed using thematic analysis, with themes mapped to the study aims.

2. Methods section

The specific qualitative approach employed must be explicitly stated to strengthen methodological transparency.

3. In Line 88 the statement “The research applied a qualitative research design” is inaccurate. Qualitative methods represent an approach rather than a design. The authors should revise this phrasing to reflect the correct methodological terminology.

REPLY: Thank you. We have changed this to: This qualitative study used a reflective thematic analysis approach to explore staff experiences of pregnancy and neonatal loss. Semi-structured individual interviews were carried out with staff working in a large NHS Trust in the UK.

4. Line 101, the comma following "short" appears misplaced and should be removed for clarity.

REPLY: Thank you. We have removed this comma.

Attachment Submitted filename: Response to reviewers R2.docx

# Editor report: Decision Letter 2

**DOI:** 10.1371/journal.pone.0358321.r005

**Authors:** Dorothy Boakye

6 Aug 2026

Thank you for submitting your manuscript to PLOS ONE. After careful consideration, we feel that it has merit but does not fully meet PLOS ONE’s publication criteria as it currently stands. Therefore, we invite you to submit a revised version of the manuscript that addresses the points raised during the review process.

plosone@plos.org. When you're ready to submit your revision, log on to https://www.editorialmanager.com/pone/ and select the 'Submissions Needing Revision' folder to locate your manuscript file.

A letter that responds to each point raised by the academic editor and reviewer(s). You should upload this letter as a separate file labeled 'Response to Reviewers'.

- A marked-up copy of your manuscript that highlights changes made to the original version. You should upload this as a separate file labeled 'Revised Manuscript with Track Changes'.
- An unmarked version of your revised paper without tracked changes. You should upload this as a separate file labeled 'Manuscript'.
- If you would like to make changes to your financial disclosure, please include your updated statement in your cover letter. Guidelines for resubmitting your figure files are available below the reviewer comments at the end of this letter.

If applicable, we recommend that you deposit your laboratory protocols in protocols.io to enhance the reproducibility of your results. Protocols.io assigns your protocol its own identifier (DOI) so that it can be cited independently in the future. For instructions see: https://journals.plos.org/plosone/s/submission-guidelines#loc-laboratory-protocols. Additionally, PLOS ONE offers an option for publishing peer-reviewed Lab Protocol articles, which describe protocols hosted on protocols.io. Read more information on sharing protocols at https://plos.org/protocols?utm_medium=editorial-email&utm_source=authorletters&utm_campaign=protocols.

As the corresponding author, your ORCID iD is verified in the submission system and will appear in the published article. PLOS supports the use of ORCID, and we encourage all coauthors to register for an ORCID iD and use it as well. Please encourage your coauthors to verify their ORCID iD within the submission system before final acceptance, as unverified ORCID iDs will not appear in the published article. *Only*  the individual author can complete the verification step; PLOS staff *cannot*  verify ORCID iDs on behalf of authors.

Please note that I have acted as Reviewer 1 for this manuscript on the original (PONE-D-26-16902 R0) and first revision of the manuscript (PONE-D-26-16902_R1)

We look forward to receiving your revised manuscript.

Kind regards,

Dorothy Serwaa Boakye, PGDE,MA,Mphil, RN, BSc

Academic Editor

PLOS One

Journal Requirements:

1. If the reviewer comments include a recommendation to cite specific previously published works, please review and evaluate these publications to determine whether they are relevant and should be cited. There is no requirement to cite these works unless the editor has indicated otherwise.

2. Please review your reference list to ensure that it is complete and correct. If you have cited papers that have been retracted, please include the rationale for doing so in the manuscript text, or remove these references and replace them with relevant current references. Any changes to the reference list should be mentioned in the rebuttal letter that accompanies your revised manuscript. If you need to cite a retracted article, indicate the article’s retracted status in the References list and also include a citation and full reference for the retraction notice.

**Additional Editor Comments:**

I have reviewed your response to Reviewer #2's comments alongside the tracked-changes manuscript. While most of the requested revisions have been made, two points in your response letter do not yet match what appears in the manuscript file, and one further formatting inconsistency was introduced during the revision. I am therefore returning the manuscript for a brief, targeted revision before it can be accepted.

1. Abstract restructuring (Reviewer #2, comment 1)

Your response states that the Design, Setting, and Population information was merged into the Methods, and that the Objective and Study Aims sections were consolidated. However, the current abstract retains separate "Design," "Setting," and "Population" subheadings in addition to a newly added Background/Objective statement, and the original "Study Aims" subheading remains, largely duplicating the objective sentence you added. As it stands, the abstract now contains more redundant structure than the previous version, rather than less.

Please revise the abstract so that it contains a single Background statement (ending in one clear objective/aim), followed by a single Methods paragraph incorporating design, setting, and population detail, followed by Results and Conclusion,  without the separate subheadings and without a duplicated aims statement.

2. Line 101 comma (Reviewer #2, comment 4)

Your response indicates the misplaced comma has been removed, but it remains in the manuscript: "...completing a short, online questionnaire or by contacting the research team directly." Please remove this comma and check the response letter against the manuscript more generally before resubmission, to avoid discrepancies of this kind.

3. Methods sentence fragment (introduced during revision of comment 3)

The terminology correction to comment 3 has been made appropriately and the qualitative approach (reflective thematic analysis) is now clearly stated. However, the edited sentence in the Methods section currently reads as a fragment: "...to explore staff experiences of pregnancy and neonatal loss. utilising Semi-structured individual interviews were carried out with staff..." Please revise this for grammatical clarity. No change to content is needed, only to sentence structure.

[Note: HTML markup is below. Please do not edit.]

Reviewers' comments:

Reviewer # 1

The abstract currently contains both an “Objective” and a “Study aims” section. These are redundant, as they essentially convey the same meaning. The authors should merge these into a single statement and instead begin the abstract with a concise background that contextualizes the study.

• The design, setting, and population details should be presented within the Methods section rather than elsewhere. Additionally, the specific qualitative approach employed must be stated.

2. Methods section

The specific qualitative approach employed must be explicitly stated to strengthen methodological transparency.

3. In Line 88 the statement “The research applied a qualitative research design” is inaccurate. Qualitative methods represent an approach rather than a design. The authors should revise this phrasing to reflect the correct methodological terminology.

4. Line 101, the comma following "short" appears misplaced and should be removed for clarity.

[NOTE: If reviewer comments were submitted as an attachment file, they will be attached to this email and accessible via the submission site. Please log into your account, locate the manuscript record, and check for the action link "View Attachments". If this link does not appear, there are no attachment files.]

To ensure your figures meet our technical requirements, please review our figure guidelines: https://journals.plos.org/plosone/s/figures

You may also use PLOS’s free figure tool, NAAS, to help you prepare publication quality figures: https://journals.plos.org/plosone/s/figures#loc-tools-for-figure-preparation.

NAAS will assess whether your figures meet our technical requirements by comparing each figure against our figure specifications.

# Author comment: Author response to Decision Letter 3

**DOI:** 10.1371/journal.pone.0358321.r006

19 Aug 2026

We thank the reviewers for their additional feedback. Looking at the clean version submitted for the second round of revisions, the below requested revisions have already been addressed. We are therefore resubmitting the clean version that was submitted for revision #2 with highlights to mark the changes requested below.

1. Methods section: The specific qualitative approach employed needs to be explicitly stated for methodological transparency, both in the abstract and the main Methods section.

REPLY: This is stated in the abstract on line 24 and in the methods section in the design and setting sub section (line 83)

2. Terminology correction (Line 88): The statement "The research applied a qualitative research design" is imprecise, as qualitative research is more accurately described as an approach rather than a design.

REPLY: As above. This is stated on line 24 and 83

Attachment Submitted filename: Response to reviewers R3.docx

# Editor report: Decision Letter 3

**DOI:** 10.1371/journal.pone.0358321.r007

**Authors:** Dorothy Boakye

31 Aug 2026

Examining the mental health support needs of NHS staff exposed to patient pregnancy and neonatal loss: a qualitative study

PONE-D-26-16902R3

Dear Dr. Harrison,

We’re pleased to inform you that your manuscript has been judged scientifically suitable for publication and will be formally accepted for publication once it meets all outstanding technical requirements.

Within one week, you’ll receive an e-mail detailing the required amendments. When these have been addressed, you’ll receive a formal acceptance letter and your manuscript will be scheduled for publication.

An invoice will be generated when your article is formally accepted. Please note, if your institution has a publishing partnership with PLOS and your article meets the relevant criteria, all or part of your publication costs will be covered. Please make sure your user information is up-to-date by logging into Editorial Manager at Editorial Manager® and clicking the ‘Update My Information' link at the top of the page. For questions related to billing, please contact billing support.

If your institution or institutions have a press office, please notify them about your upcoming paper to help maximize its impact. If they’ll be preparing press materials, please inform our press team as soon as possible -- no later than 48 hours after receiving the formal acceptance. Your manuscript will remain under strict press embargo until 2 pm Eastern Time on the date of publication. For more information, please contact onepress@plos.org.

Kind regards,

Dorothy Serwaa Boakye, PGDE,MA,Mphil, RN, BSc

Academic Editor

PLOS One

Additional Editor Comments (optional):

Reviewers' comments:

# Editor report: Acceptance letter

**DOI:** 10.1371/journal.pone.0358321.r008

**Authors:** Dorothy Boakye

PONE-D-26-16902R3

PLOS One

Dear Dr. Harrison,

I'm pleased to inform you that your manuscript has been deemed suitable for publication in PLOS One. Congratulations! Your manuscript is now being handed over to our production team.

At this stage, our production department will prepare your paper for publication. This includes ensuring the following:

* All references, tables, and figures are properly cited

* All relevant supporting information is included in the manuscript submission,

* There are no issues that prevent the paper from being properly typeset

You will receive further instructions from the production team, including instructions on how to review your proof when it is ready. Please keep in mind that we are working through a large volume of accepted articles, so please give us a few days to review your paper and let you know the next and final steps.

Lastly, if your institution or institutions have a press office, please let them know about your upcoming paper now to help maximize its impact. If they'll be preparing press materials, please inform our press team within the next 48 hours. Your manuscript will remain under strict press embargo until 2 pm Eastern Time on the date of publication. For more information, please contact onepress@plos.org.

You will receive an invoice from PLOS for your publication fee after your manuscript has reached the completed accept phase. If you receive an email requesting payment before acceptance or for any other service, this may be a phishing scheme. Learn how to identify phishing emails and protect your accounts at https://explore.plos.org/phishing.

If we can help with anything else, please email us at customercare@plos.org.

Thank you for submitting your work to PLOS One and supporting open access.

Kind regards,

PLOS One Editorial Office Staff

on behalf of

Dr. Dorothy Serwaa Boakye

Academic Editor

PLOS One
